# Randomization Modeling to Ascertain Clustering Patterns of Human Papillomavirus Types Detected in Cervicovaginal Samples in the United States

**DOI:** 10.1371/journal.pone.0082761

**Published:** 2013-12-18

**Authors:** Troy David Querec, Brian Mohan Gurbaxani, Elizabeth Robinson Unger

**Affiliations:** Chronic Viral Diseases Branch, Division of High-Consequence Pathogens and Pathology, Centers for Disease Control and Prevention, Atlanta, Georgia, United States of America; Georgetown University, United States of America

## Abstract

Detection of multiple human papillomavirus (HPV) types in the genital tract is common. Associations among HPV types may impact HPV vaccination modeling and type replacement. The objectives were to determine the distribution of concurrent HPV type infections in cervicovaginal samples and examine type-specific associations. We analyzed HPV genotyping results from 32,245 cervicovaginal specimens collected from women aged 11 to 83 years in the United States from 2001 through 2011. Statistical power was enhanced by combining 6 separate studies. Expected concurrent infection frequencies from a series of permutation models, each with increasing fidelity to the real data, were compared with the observed data. Statistics were computed based on the distributional properties of the randomized data. Concurrent detection occurred more than expected with 0 or ≥3 HPV types and less than expected with 1 and 2 types. Some women bear a disproportionate burden of the HPV type prevalence. Type associations were observed that exceeded multiple hypothesis corrected significance. Multiple HPV types were detected more frequently than expected by chance and associations among particular HPV types were detected. However vaccine-targeted types were not specifically affected, supporting the expectation that current bivalent/quadrivalent HPV vaccination will not result in type replacement with other high-risk types.

## Introduction

Genital HPV is the most common sexually transmitted infection [1], [2]. HPV is a necessary, but not sufficient, cause of cervical cancer [3], [4] and genital warts [5] and is associated with other anogenital cancers [6]. Of the over 100 HPV types, at least 40 infect the anogenital tract. Twelve types have evidence sufficient for classification as “high-risk” HPV (HR HPV) and an additional 13 have some limited evidence of cancer risk [7]. Concurrent infection with multiple HPV types is common, especially in young women and in people with HIV infections [8]–[16]. Concurrent infection with multiple HPV types compared to single HR-HPV infection has been found to increase the risk of disease in some reports [17], [18] but not in others [4], [15], [19]. The high prevalence of HPV and frequency of concurrent infections with more than one type provides an opportunity for HPV type interactions.

The current HPV vaccines target the two HR-HPV types (HPVs 16 and 18) associated with 70% of cervical cancers. If, however, types display positive associations to inflate infection rates, broad HPV vaccination coverage may lead to reduction of HPV types not targeted by the vaccine, i.e. “cross-protection” not based on cross-reaction immunity but as a result of reduced fitness of positively associated types. Alternatively, negative associations among types may lead to type replacement of non-vaccine types as competing types targeted by vaccines are reduced [20].

Associations among multiple HPV types have been examined in prior studies, but the conclusions are contradictory [21]–[28]. A limiting factor for robust analysis of type associations is the number of HPV positive samples in a dataset relative to the hundreds of potential type combinations.

The aim of the present study is to address overall and type-specific HPV associations by taking advantage of a large laboratory database of HPV results obtained using the same validated HPV typing assay. Aggregating multiple study datasets provides greater statistical power in analyzing potential HPV type combinations. We employed a permutation methodology to test first a complete null model of random type association, and then gradually less naïve models with preserved higher orders of data structure [29].

## Materials and Methods

### Dataset

The dataset includes anonymized HPV typing results from 32,245 cervicovaginal samples from six studies of women aged 11 to 83 years conducted between 2001 through 2011 (Table 1). Because all data were rendered non-identifiable before this analysis was conceived, the project does not involve human subjects under United States Department of Health and Human Services' Code of Federal Regulations Title 45 Section 46.102(f). All samples were from different immune-competent women in the United States. The majority of samples were clinician collected exfoliated cervical cells – 15,086 ThinPrep® (Hologic, Bedford, MA, USA), 10,147 Specimen Transport Medium™ (Qiagen, Valencia, CA); the remainder was self-collected cervicovaginal specimens. Most specimens (28,417) were from screening or general populations, but 3,828 were from colposcopy clinics. A binary matrix of the HPV typing results with the general versus colposcopy population status has been made available online (Data S1).

**Table 1:** Summary of the studies in the aggregate analysis.

Study	1	2	3	4	5	6
**Population**	General	Colposcopy	Colposcopy	General	General	General
**Collection Method**	Clinical Specimen Transport Medium™	Clinical ThinPrep®	Clinical ThinPrep®	Clinical ThinPrep®	Clinical ThinPrep®	Self-Collected Swab
**Tissue**	Cervical	Cervical	Cervical	Cervical	Cervical	Cervical-Vaginal

HPV Types per Person	Number of People						k Strata Totals
**0**	6639	503	175	637	6547	4015	18516
**1**	1664	598	592	232	1901	1384	6371
**2**	965	310	531	110	892	768	3576
**3**	477	174	373	74	410	378	1886
**4**	225	74	205	37	206	199	946
**5**	85	23	122	15	107	118	470
**6**	51	12	49	7	40	71	230
**7**	21	7	44	4	21	41	138
**8**	11	3	13	1	8	17	53
**9**	4	0	10	1	6	11	32
**10**	3	1	3	0	2	8	17
**11**	1	0	2	0	0	0	3
**12**	0	1	1	0	0	1	3
**13**	0	0	1	0	0	1	2
**14**	1	0	1	0	0	0	2
**Study Totals**	10147	1706	2122	1118	10140	7012	32245

### HPV DNA Genotyping

All samples were extracted to yield DNA or total nucleic acids (DNA and RNA), and 0.5–1% of the total sample extract was tested for HPV. HPV typing was performed using the Linear Array HPV Genotyping Test (LA, Roche Diagnostics, Indianapolis, IN) according to the manufacturer's protocol. The LA detects 37 HPV types (6, 11, 16, 18, 26, 31, 33, 35, 39, 40, 42, 45, 51, XR(52), 53, 54, 55, 56, 58, 59, 61, 62, 64, 66, 67, 68, 69, 70, 71, 72, 73, 81, 82, 83, 84, 89, IS39). HPV types 33, 35, and 58 have type-specific probes, but HPV 52's probe (XR) also reacts with HPV 33, 35, and 58. Therefore when both a HPV 33, 35, or 58 probe and the XR probe is positive, the LA result is equivocal as to whether HPV 52 is being detected or the probe is just reacting with HPV 33, 35, or 58. Samples with equivocal results for HPV 52 were tested with a quantitative type-specific assay for HPV 52 [30]. All HPV DNA testing was conducted in a single laboratory with rigorous quality control and high reproducibility.

### HPV 56 and 66 Genotyping Specificity

In order to test the type specificity of the LA for HPVs 56 and 66, high copy numbers of their L1 genes were amplified from plasmids. The primers were designed to flank the LA target region (Table S1). Three different primer sets for each HPV type were tested for efficiency of amplifying the target region and reproducibility of LA specificity. After PCR product quantification, 10 ul of each amplicon was directly incorporated into the LA assay.

### Statistical Analysis

#### Generating Expected Models

**Figure 1:**
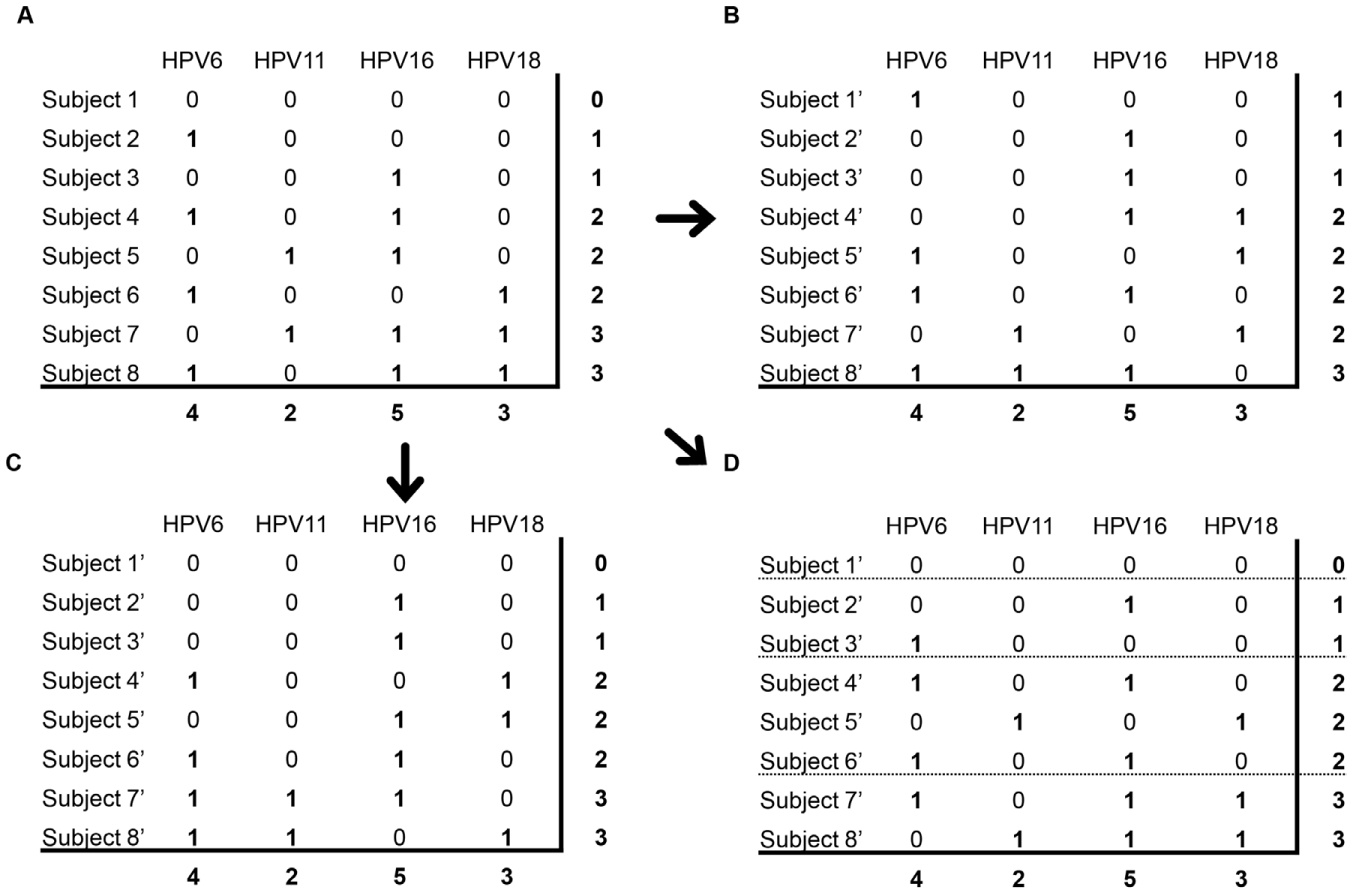
An abbreviated example of matrix randomizations. The observed data (**A**) of HPV infection status is organized in binary matrix indicating type-specific infection status. To generate expected models, data are randomized while preserving column sums (null model; **B**) or row and column sum (non-strata model; **C**). Additionally data were stratified by HPV types per person (k-strata model 2; **D**) or/and study data source (study and study-k strata models; not shown) with randomization restricted to within each strata.

To determine the expected number of concurrent infections, the matrix of observed results was randomized in five different ways, depending on the characteristics of the observed data being controlled, using R (Figure 1 and Table S2) [31]. Observed data had rows by subject and columns by 37 HPV types with zeros (0 s) indicating negative HPV results and ones (1 s) indicating positive (Figure 1A). For the null model of complete random association of HPV types in Figure 1B, the 0 s and 1 s were shuffled within each column using the ‘sample’ function of the R *base* package version 2.14.1, preserving the count of each HPV type (fixed column sums) while allowing the number of types per subject to vary (variable row sums).

For the subsequent matrix randomizations, the ‘permatswap’ function created a series of matrix permutation models, each with increasing fidelity to the real data, where some of the higher order data structure is preserved during the randomization [32]. The trial-swap method overcomes the deficiencies of other fixed column/row sum algorithms in avoiding biased randomized matrices [33]. The number of randomization steps was set to 5×10^7^ for the ‘burnin’ parameter as this value maximized Bray-Curtis dissimilarity values, indicating effective matrix randomization [33]. In the non-strata naïve model, the matrix was randomized while preserving the marginal HPV type counts and types per person (fixed row and column sums; Figure 1C). For higher fidelity randomization models, observations were stratified based on the 6 studies (study strata), 1 through 14 concurrent infections (k strata), or a combination of both (study-k strata) using the ‘strata’ parameter of ‘permatswap’. Randomization within each strata had fixed column and row sums (Figure 1D). To assess general trends of concurrent infections, 10,000 randomized matrices were generated from all the subjects (HPV positive and negative) and compared to the expected results. To assess the significance of specific type combinations, 1,000 randomized matrices were generated from the HPV positive subjects and analyzed as discussed below.

#### Calculating Statistics from Expected Models

Counting occurrences of type combinations, whether in the observed data or in the randomized matrices for a given model, was done in Perl (Active Perl 5.8; ActiveState, Vancouver, BC). A key feature of the counting, whether in observed or permuted data, was that specific type combinations were counted whether or not additional types were present. The results for the randomized matrices were then matched to type combinations observed in the real data.

Results for the Perl scripts above were then read into a Mathematica program to assess statistical significance (Wolfram Research, Champaign IL). First, the expected counts of any given type combination in the permutation models were fit to a Poisson probability density function (pdf; Figure S1A). The Poisson distribution fits were consistently very good (model p>0.99). To graphically indicate significant type combinations, the observed counts were compared to p-value boundaries created from the Poisson distribution and the mean value parameter for each type combination generated in the permutation models (recall a Poisson distribution requires only the single parameter). Type combination counts corresponding to p-value boundaries of 10^−4^, 10^−6^, 10^−8^, etc. were calculated for both the right (observed > expected) and left (observed < expected) tails of the Poisson distribution. Observed counts were then plotted against expected counts for a given permutation model so that type combinations falling outside the boundary value lines could be easily seen. Besides the hypothetical p-value boundaries, actual p-values and Z-scores were computed for each specific type combination using the Poisson fit mean values from the permutation runs. Because the type combination frequencies observed in the 1,000 randomized matrices precisely fit a known and well characterized distribution, p-values <0.001 can be reliably estimated.

Due to the number of HPV type combinations analyzed, the Benjamini and Hochberg false-discovery rate (fdr) was calculated using ‘p.adjust’ in R to control for spurious results [34].

Further details on calculating the statistics are in Methods S1.

All code (R, Perl, or Mathematica) is available upon request.

## Results

Of 32,245 subjects, 13,729 were positive for ≥1 HPV type, and 7,358 were positive for multiple HPV types (Table 1). Allowing for the subset of individuals with multiple HPV types, specific HR HPV types were detected 15,780 times out of a total of 28,666 HPV type positive results.

### General Trends of Concurrent Infections

**Figure 2:**
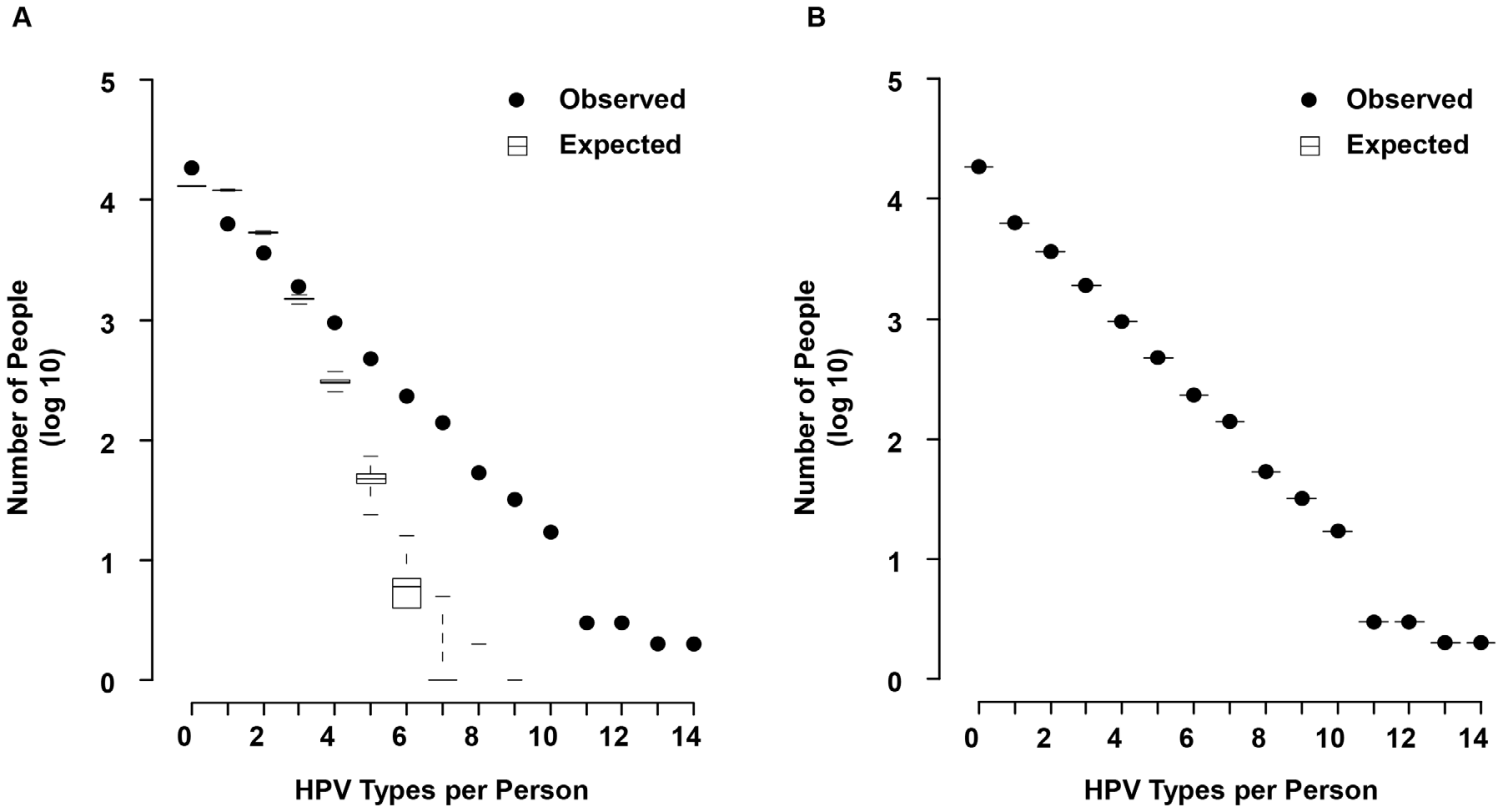
Differences between observed and expected number of people with concurrent infections. Dots representing observed number of concurrent infections are plotted against boxplots of expected distribution from 10,000 randomized matrices. Boxplots indicate the minimums and maximums, 25% and 75% quartiles, and medians of randomized matrices. (**A**) tests the null model of complete random association of HPV types as in Figure 1B. (**B**) controls for observed types per person in the non-strata fixed margins permutation model as in Figure 1C. Note that because the margins for all randomized matrices are set to be equal to that of the observed data and thus the same value for each permutation, the “boxes” have no ranges and are represented as single-value lines.

Figure 2A shows the results of testing the null model of complete random association among HPV types as diagramed in Figure 1A and B. The observed data did not overlap with the expected box plot, indicating the observed concurrent infections of the aggregate dataset do not fit an unstructured random assortment. The box heights in Figure 2A reveal that all of the 10,000 iterations of the null expected model are highly self-consistent for the number of HPV types per person and highly inconsistent with the real data. At the maximum number of 9 expected HPV types per person in the null model, only 2 people out of 3.22×10^8^ (3.22×10^4^ people/iteration × 1×10^4^ iterations) are expected to have this many concurrent HPV types detected, whereas the observed data contains a continuous distribution of subjects with up to 14 HPV types detected at the same time. Furthermore, infection with 0 and 3 through 14 HPV types occurred more than expected by the null model. In contrast, infection with 1 and 2 HPV types occurred less than expected.

Because such differences in general trends between the observed data and null model would bias the analysis of specific HPV type associations, these trends were preserved in subsequent randomizations to create continuously less naïve models. This randomization resulted in the distribution of each of the 10,000 permutation matrices exactly matching the observed results (non-strata model; Figures 1C and 2B). However dividing the data into 13 HR and 24 LR HPV types post-randomization shows that the observed data has an additional level of structure which may confound specific type analysis; the general distribution of HR and LR HPV types differ between the observed and this first permutation model (Figure S2A). Compared to this model, the HR types are either alone or with 1 other HPV type more than expected. Conversely, the LR types tend to have more observations in high numbers of concurrent infections than expected. Grouping the dataset by studies, the differences in distribution among HR and LR HPV types are blurred in the general populations but increase further in the colposcopy populations (Figure S2B and C). To control for this level of structure, randomizations are conducted by conserving the type prevalence and HPV types per person within each strata of HPV types per person (k strata model; Figure 1D). In other words, data are randomized separately within people with 2 types, people with 3 types, etc. This eliminates any difference in the distribution of HR and LR HPV or even each specific HPV type k within strata between the observed data and model. This method of stratification removes 6,371 subjects with only 1 detected HPV type from the concurrent infection analysis, and limits the analysis to the 7,358 subjects with ≥2 concurrent HPV types. Additionally data are stratified by the study data source (study strata) and by combined study and types per person (study-k strata). For this final and least naïve study-k model of the 84 possible strata (6 studies ×14 k strata), 67 strata exist in the data as some studies do not have all 14 k strata. For the study-k model, each observation of an HPV type is restricted to potential interactions only with other types of that stratum. The association among HPV types is still randomized within these imposed structures.

### Significant HPV Type Combinations

**Figure 3:**
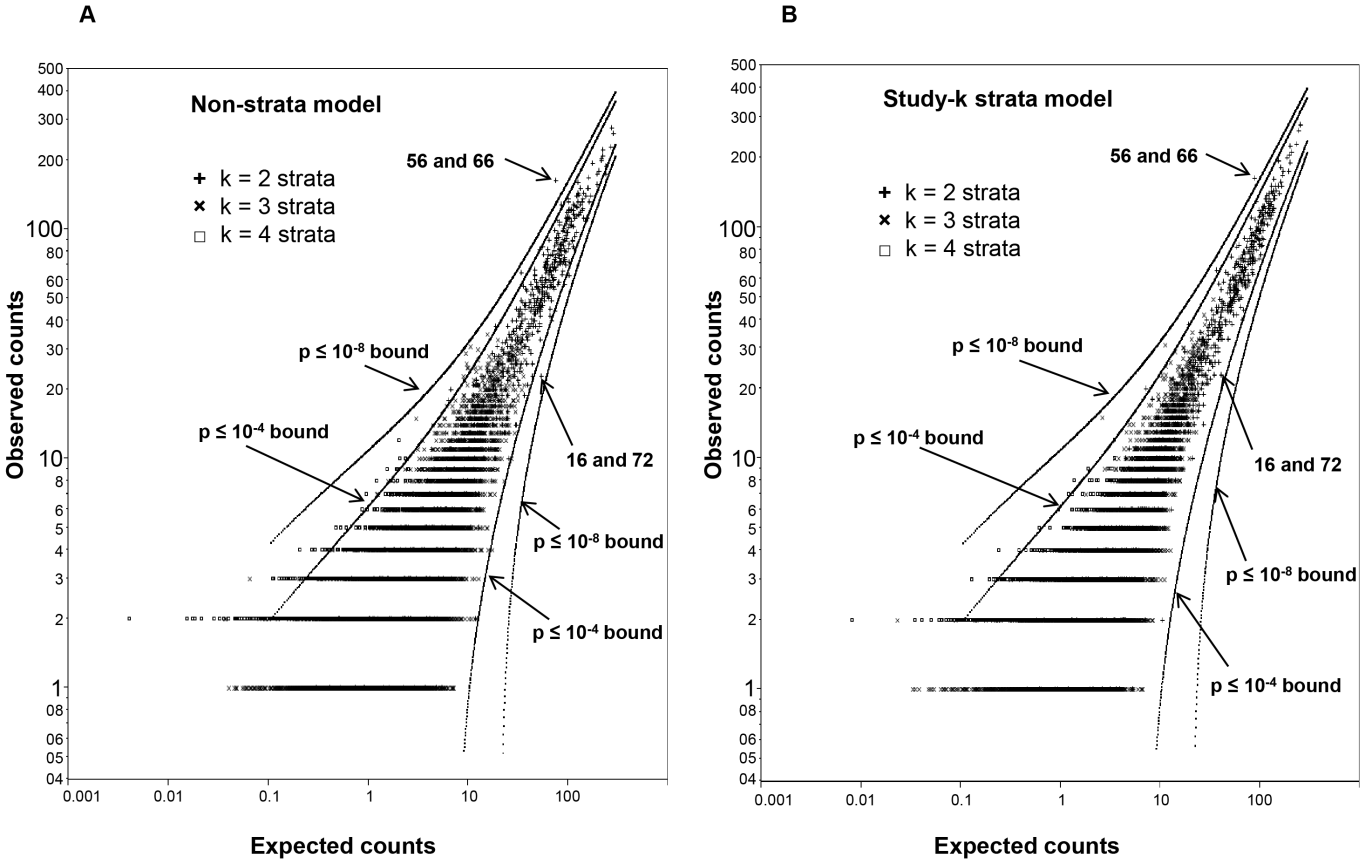
Significant combinations for k =  two, three and four HPV types. The graphs depict the non-strata (**A**) and study-k (**B**) permutation models. Boundary lines (dots) show p-value significance at 10^−4^ and 10^−8^. The mean expected counts on the x-axis represents the average number of observations from 1,000 randomized matrices; the y-axis shows the actual observed counts for the type combinations. Single observations of 4 HPV type combinations are excluded.

The permutation models were used to determine which specific HPV type combinations are truly unexpected and which combinations are artifacts of study factors structuring the dataset. Figure 3 plots the observations for combinations of 2 through 4 HPV types for (A) the most naïve model (the non-strata model with only row and column sums preserved) and (B) the least naïve model (the study-k strata model). The significance boundary lines for under observed type combinations bottom out on the right side of the plot. This is because even zero observations of most 4 type combinations can be expected and p = 0.0001 is difficult to achieve even in a dataset of this size. The pairs with the largest positive and negative Z-scores, 56 and 66 on the over observed side (left side of Figure 3) and 16 and 72 on the under observed side (right side), are indicated for illustration. Very few under observed 3 and 4 type combinations were detected.

Table 2 lists HPV pairs observed more or less than expected in the various models. As the general distribution of the null model in Figure 2 differed so drastically from the observed results, a list of significant specific type combinations for this model is uninformative and therefore not included. Of the permutation models, the non-stratified model differs the most from the observed results, and therefore finds the greatest number of HPV pairs to be significant (47 pairs). However as additional levels of data structures are conserved in the less naïve models, fewer HPV pairs are significant. Furthermore, only HPV pairs observed more than expected passed the stringency test of the highest fidelity models; no HPV pairs were significantly under observed against the k and study-k models. However for the non-strata and study strata models, vaccine-targeted type HPV 16 was found to be observed less than expected with HPVs 58, 62, 69, 70, and 72. Several of the HPV pairs observed more than expected were within the same species. The association of HPVs 56 and 66 from species α6 has the highest z-score compared to all models with a z-score indicating at least 8 standard deviations away from the model mean.

**Table 2:** Significant HPV Pairs.

A Observed/Expected (Z-score) for Models.						
HPV Types*	Species	Observations	Non-Strata	Study Strata	k Strata	Study-k Strata
**56,66**	α6, α6	164	2.2 (10.4)	2.1 (9.9)	1.8 (8.0)	1.9 (8.1)
**51,82**	α5, α5	65	2.0 (5.5)	2.0 (5.6)	1.8 (4.9)	1.8 (4.8)
**51,69**	α5, α5	20	3.0 (5.2)	2.9 (5.0)	2.7 (4.7)	2.8 (4.8)
**33,58**	α9, α9	38	2.0 (4.5)	1.7 (3.2)	2.2 (5.0)	2.0 (4.3)
**52,58**	α9, α9	129	1.5 (4.8)	1.4 (4.0)	1.5 (5.0)	1.4 (4.0)
**51,IS39**	α5, α5	18	2.9 (4.7)	2.7 (4.4)	2.2 (3.4)	2.2 (3.4)
**16,18**	α9, α7	194			1.3 (3.7)	1.3 (3.3)
**31,45**	α9, α7	75			1.5 (3.3)	1.5 (3.3)
**62,83**	α3, α3	110	1.4 (3.7)	1.4 (3.2)	1.4 (3.5)	1.4 (3.2)
**55,62**	α10, α3	82	1.6 (4.2)	1.5 (3.6)	1.5 (3.6)	1.4 (3.1)
**81,83**	α3, α3	57	1.6 (3.5)		1.6 (3.5)	1.5 (3.0)
**62,81**	α3, α3	84	1.5 (3.8)		1.5 (4.0)	
**71,72**	α15, α3	11	2.6 (3.2)		3.2 (4.0)	
**72,83**	α3, α3	29	1.7 (2.8)		1.9 (3.6)	
**61,62**	α3, α3	133	1.3 (3.4)		1.4 (3.5)	
**62,70**	α3, α7	63			1.5 (3.4)	
**61,70**	α3, α7	54			1.6 (3.3)	
**61,83**	α3, α3	90	1.4 (3.3)		1.4 (3.0)	
**70,72**	α7, α3	15			2.1 (2.9)	
**71,83**	α15, α3	22	1.9 (2.9)		1.8 (2.8)	
**67,84**	α9, α3	64			1.4 (2.8)	
**62,72**	α3, α3	35			1.6 (2.8)	
**61,71**	α3, α15	24			1.7 (2.8)	
**16,39**	α9, α7	224			1.2 (2.7)	
**66,89**	α6, α3	137	1.4 (3.7)	1.3 (3.5)		
**6,89**	α10, α3	99	1.4 (3.0)	1.4 (3.1)		
**51,66**	α5, α6	161	1.3 (3.3)	1.3 (3.0)		
**35,83**	α9, α3	67	1.5 (3.1)			
**54,61**	α13, α3	108	1.3 (3.0)			
**11,18**	α10, α7	21	1.9 (3.0)			
**42,56**	α1, α6	78	1.4 (2.9)			
**26,35**	α5, α9	13	2.2 (2.9)			
**42,73**	α1, α11	55	1.5 (2.8)			

B Observed/Expected (Z-score) for Models						
HPV Types*	Species	Observations	Non-Strata	Study Strata	k Strata	Study-k Strata
**16,58**	α9, α9	109	0.7 (−3.1)	0.7 (−3.9)		
**16,72**	α9, α3	23	0.4 (−4.1)	0.5 (−3.7)		
**31,62**	α9, α3	63	0.6 (−3.8)	0.7 (−3.3)		
**58,59**	α9, α7	40	0.6 (−3.3)	0.6 (−3.3)		
**66,70**	α6, α7	23	0.5 (−3.1)	0.5 (−3.1)		
**66,72**	α6, α3	10	0.4 (−3.0)	0.4 (−3.1)		
**16,62**	α9, α3	175	0.7 (−4.0)	0.8 (−2.9)		
**16,70**	α9, α7	58	0.6 (−3.5)	0.7 (−2.9)		
**39,83**	α7, α3	44	0.6 (−3.4)	0.7 (−2.9)		
**51,72**	α5, α3	14	0.5 (−2.9)	0.5 (−2.9)		
**31,83**	α9, α3	42	0.6 (−2.9)	0.6 (−2.8)		
**16,69**	α9, α5	2	0.2 (−2.8)	0.2 (−2.8)		
**53,81**	α6, α3	45		0.7 (−2.8)		
**16,83**	α9, α3	113	0.7 (−3.2)			
**16,84**	α9, α3	190	0.8 (−3.0)			
**67,83**	α9, α3	14	0.5 (−2.9)			
**16,55**	α9, α10	72	0.7 (−2.9)			
**31,70**	α9, α7	21	0.5 (−2.9)			
**16,71**	α9, α15	19	0.5 (−2.9)			
**39,62**	α7, α3	83	0.7 (−2.8)			
**39,81**	α7, α3	33	0.6 (−2.7)			
**39,84**	α7, α3	84	0.7 (−2.7)			
**39,70**	α7, α7	26	0.6 (−2.7)			
**16,61**	α9, α3	157	0.8 (−2.7)			

≤0.05 for (**A**) observed more than expected and (**B**) observed less than expected. Results are listed for HPV pairs with fdr

*HR-HPV types are underlined.

The data accommodate a more detailed scrutiny of multiple concurrent infections. The significant results for 2, 3, and 4 type combinations are interdependent (Tables 2A, S3, S4, and S5). Although significantly over observed pairs like HPV types 56 and 66 pickup other types and therefore may drive the significance for 3 and 4 type combinations, the possibility remains that real synergies exist between 3 or more types, or they appear to exist because of cross-hybridizing types.

Because HPV 56 and 66 are strongly associated with each other and are closely related genetically, it is important to exclude artifacts of the assay that could account for the association. We tested the specificity of the genotyping assay for these two types using high copy numbers of type-specific templates. PCR amplified HPV DNA only hybridized to the intended probe band without cross hybridization (Figure S3).

## Discussion

Observations from 6 different HPV genotyping studies were combined to address whether concurrent HPV infections vary from random assortment. Observations were compared with both a null and progressively less naïve models as row and column sums, particular study, and concurrent infection burden variables in the dataset were incorporated. The results of our null model are consistent with other reports of multiple infections being detected more than expected [21]–[25], [27], [28], [35]. In our permutation models, which incorporate restrictions based on the observed data structure, certain specific HPV type combinations are statistically significantly over or under observed. These models have greater utility in testing the validity of alternative hypotheses and serve as a more rigorous control than null models [36]. Additionally we have demonstrated a novel approach to combine data from multiple datasets that preserves statistical rigor while enhancing statistical power.

Other groups have noted excess concurrent HPV type infections compared to null models. The null hypotheses included maximum-likelihood based on an assumed Poisson distribution [35], Hardy-Weinberg equilibrium test based calculation [23], simulating concurrent infections based on type frequencies in populations [21], [24], and Bayesian logistic regression [27], [28]. These null models demonstrate that HPV types are not distributed purely at random in populations and are in agreement with the results using our null model. Some women are infected with high numbers of multiple HPV types and bear a disproportionate burden of HPV types compared to the general population.

Some authors have controlled for risk factors of multiple HPV infections while analyzing type-specific interactions. People infected with HPV are more likely to acquire additional HPV types than are uninfected persons [37]. Factors include age, immunity, smoking, cervical pathology, lifetime number of sexual partners, frequency of sexual intercourse, fidelity of sexual partner, and study area.[8]–[16] Controlling for subsets of these factors has reduced the discrepancies between the observed data and expectations in various models [24], [27], [28]. However there are limits in the ability to fully control for all known risk factors even if they are measured, and the potential impact of unknown factors cannot be addressed.

This current analysis takes a different approach to test HPV type associations. The matrix permutation models presented in this manuscript are based on recommendations as discussed by Schwab, et al. [36]. Our models do not incorporate knowledge about underling processes or confounding factors for multiple HPV infections. They attempt a more rigorous challenge than the null model by randomizing the data within basic data structure restrictions. By fixing row sums and stratifying on k-strata, the number of HPV types per person is accounted for regardless of knowing or not knowing the prevalence of risk factors for multiple HPV infections in the study populations. This increases the number of suitable datasets. Instead of being restricted to data in which at least most of the risk factors are recorded, all datasets with sound laboratory assays of type-specific HPV results are applicable. The tested HPV types can even vary among the studies.

Another limitation of the previous work is the restriction of each analysis to single study datasets. Prior studies have relatively small specimen numbers compared to the number of possible HPV combinations. Often, HPV testing was performed for a small subset of genital HPV types. Even with multiple HPV types assayed, because of small sample size, statistical analysis was limited to a few HPV types. In the largest previous study with 13,961 women, only 1,451 were HPV positive [27]. In contrast our current dataset has 13,729 HPV positive subjects of which 7,358 are positive for multiple HPV types. While combining multiple datasets results in greater statistical power due to increased sample size, the heterogeneity of the aggregated studies is a complicating factor. The studies in our analysis varied by population, collection method, and target tissue. A model randomized across studies increases the risk of type I error. We address this issue by stratifying the aggregate dataset by study and confining the randomization to within each stratum. Consistent with weeding out type I errors, significant type combinations were reduced in the study strata model compared to the non-strata model.

The approach we used gives greater confidence in interpreting the biologic significance of the identified type associations. Pairs of HPV types detected more frequently than expected were often from the same species [38]. HPVs 56 and 66 are from species α6. HPVs 51, 69, 82, and IS39 are all from species α5, and IS39 is considered a subtype of HPV 82. Finally HPVs 33, 35, 52, and 58 are part of α9. Probes for HPV 52 are known to cross hybridize with HPVs 33, 35, and 58. Vaccarella et al. also found positive association among α9 types 33, 35, 52, and 58, but attributed this to the ambiguity of HPV 52 in the enzyme immunoassay [27] and line blot assay [28]. For the line blot assay used for our data, whenever the HPV 52 probe and any of these other 3 probes are positive, the line blot assay is ambiguous for detection of HPV 52. Our studies used a subsequent type-specific PCR assay for HPV 52 to eliminate the ambiguity in these situations. Because of the genetic similarity within species, the limits of type-specificity in consensus PCR assays (such as LA) due to cross-hybridization could be suspected. However all results were obtained under stringent quality control, and high copy numbers of HPV 56 and 66 DNAs did not cross hybridize. Uncharacterized HPV types that produce amplicons that hybridize to multiple probes cannot be completely ruled out but are very unlikely because of the design of the assay and previous validation studies. Positive associations among HPV types were also found between non-species types. The significant type combinations likely indicate a genuine association among the above species types.

Fewer and less significant negative associations among HPV types were identified. No pairs of HPV types passed the statistical cutoffs for the stringent k strata and study-k strata models, and no negative associations for any model were found for combinations of 3 and 4 types. HPV 16 was frequently included as one of the types of pairs observed less frequently than expected in the non-strata and study strata models. The types observed less frequently than expected with HPV 16 are candidates for type replacement following reduction of HPV 16 by vaccination. The only HR type in this group was HPV 58; most negative associations with HPV 16 were with LR types. A nonavalent HPV vaccine in clinical trials is formulated to target HPV 6, 11, 16, 18, 31, 33, 45, 52 and 58, and thus HPV 58 would be targeted and at reduced risk of replacing HPV 16. Also, the study strata model indicates a possible negative association between HR types 58 and 59. However no negative associations were significant against the least naïve models, reducing the probability of type replacement.

A strength of the implemented method is that distribution functions were tightly fit to the permutated data to accurately calculate p-values. Typically for permutated data, p-values can only be calculated down to the inverse of the number of permutations. If a HPV combination does not occur even once at of 1000 permutations, the p-value would be <0.001. However because a distribution function can be fit to the permutated data, we can calculate p-values below the permutation limit. Thus p-values down to 10^−8^ and smaller can be calculated without needing 10^8^ permutations. This allows accurate significance testing while conserving computer resources.

Limitations exist with the current analysis. The contribution of each study to the analysis is proportional to the sample size of each study. Thus larger studies contribute more to the final results. A weighting factor for each study stratum would adjust this. We suggest that imposing the structure of the observed data in terms of column and row sums, and stratification, controls for the risk factors of multiple HPV types, e.g. risk factors for increasing HPV exposure. However without these variables in the dataset, this proposition cannot be tested directly.

The methodology presented can be applied to other data. Indeed, the current analysis is an expansion of a previous application. This randomization within strata was first used to test species' associations on archipelago islands [29]. By stratifying the dataset prior to randomization within the strata, it relaxes the analyst from making assumptions about the null distribution of the data and having information available on risk factors. By stratifying on studies, this methodology may be adapted to prospectively analyzing data from meta-anaylsis studies.[39] The presented analysis consisted of a binary matrix, however the algorithms used can be applied to other data types, expanding its application [32].

We have presented a novel approach to HPV concurrent infection analysis, which has allowed us to obtain greater statistical power to address the question of HPV type association as well as provide new methods to analyze aggregate datasets.

## Supporting Information

Figure S1 **Illustration of how 1000 randomized matrices are used to determine significance boundaries shown in** **Figure 2** **.** Example (**A**) of a Poisson probability density function fit to the observed counts for a given type combination in 100 iterations of a permutation model Monte Carlo randomization. The plot (**B**) shows the number of observations needed to meet p = 0.0001 significance (y-axis) vs. number expected by a given permutation model (x-axis). The x-axis corresponds to the mean number of counts observed in the 1000 Monte Carlo runs from any of the models. The equation embedded shows the power law fit used to create the boundary value lines on the left side of the plot (i.e. over observed type combinations) in Figure 3. The fits describe the right tail of the Poisson pdf (the number of observations needed to match the level of significance) fit to the mean number of observations for any given type combination in the Monte Carlo runs. For example, if 2^8^ = 256 counts are expected in the given permutation model, then roughly 340 observations in the real data are needed to satisfy p = 0.0001. If only 16 counts are expected, the needed number observed counts increases (as a ratio relative to expected) to about 40 for the same level of significance. Rarely observed type combinations with very rare expected values in the permutation models (i.e. candidates for over observed combinations, not under observed) were excluded from the analysis of significance. This is because, for example, it is difficult to determine the true significance or impact of a 4 type combination that is seen in the observed data only once, even if it was expected <0.001 times (for a very large observed/expected ratio) in a database of 30,000 specimens.
(TIF)

Figure S2 **Difference between the observed data and the 10,000 non-strata model segregated by high-risk and low-risk HPV types:** all subjects (**A**), general population (**B**), colposcopy population (**C**).
(TIF)

Figure S3 **Testing specificity of HPV DNA genotyping test** on PCR amplified plasmid DNA for (**A**) HPV 56 and (**B**) 66.
(TIF)

Table S1 **Primer sequences used for HPV 56 and 66 PCR amplification.**
(DOC)

Table S2 **Key parameters and assumptions of the different randomization models.**
(DOC)

Table S3 **Significant 3 HPV type combinations.**
(DOC)

Table S4 **Significant 4 HPV type combinations.**
(DOC)

Table S5 **Number of times HPV types appeared in significant combinations.**
(DOC)

Methods S1 **Supplementary methods for statistical calculations.**
(DOC)

Data S1 **Supplementary data.**
(TXT)
