# Supplementary figures and images for: Randomization Modeling to Ascertain Clustering Patterns of Human Papillomavirus Types Detected in Cervicovaginal Samples in the United States
Source: PLoS One. 2013 Dec 18;8(12):e82761. doi: 10.1371/journal.pone.0082761 (PMC3867389; doi:10.1371/journal.pone.0082761)

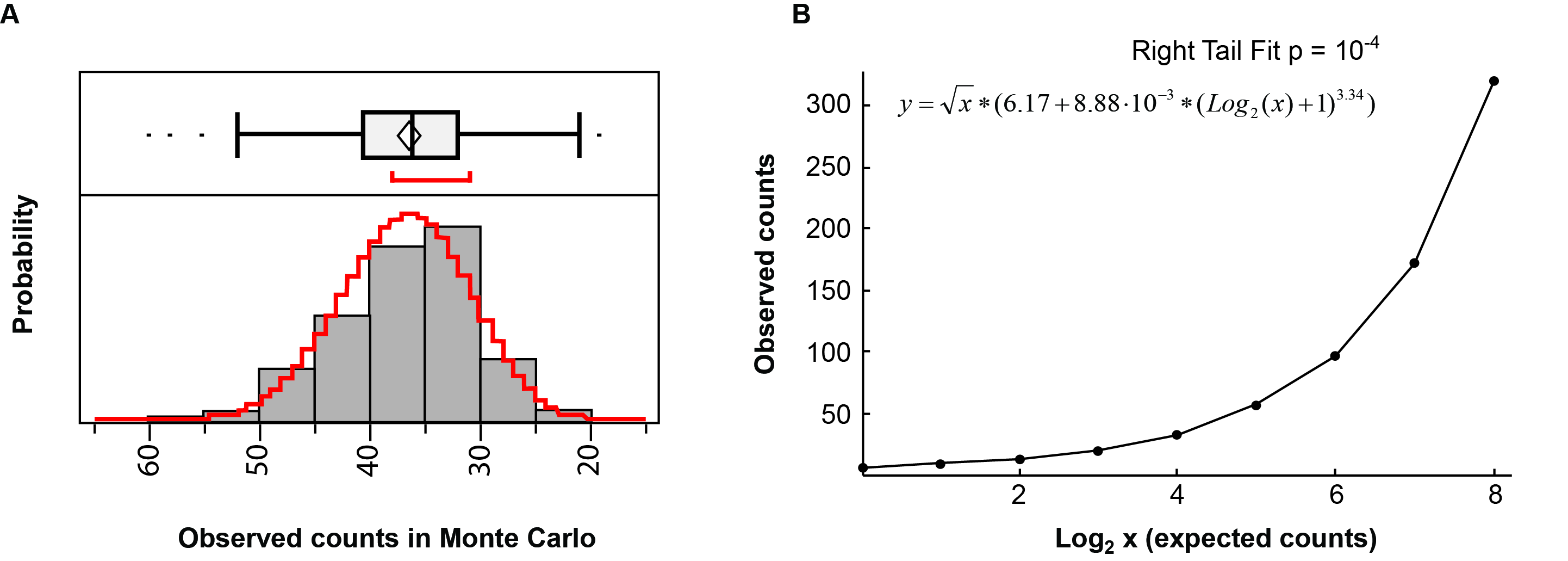

Supplement: Figure S1 — Illustration of how 1000 randomized matrices are used to determine significance boundaries shown in Figure 2 . Example (A) of a Poisson probability density function fit to the observed counts for a given type combination in 100 iterations of a permutation model Monte Carlo randomization. The plot (B) shows the number of observations needed to meet p = 0.0001 significance (y-axis) vs. number expected by a given permutation model (x-axis). The x-axis corresponds to the mean number of counts observed in the 1000 Monte Carlo runs from any of the models. The equation embedded shows the power law fit used to create the boundary value lines on the left side of the plot (i.e. over observed type combinations) in Figure 3. The fits describe the right tail of the Poisson pdf (the number of observations needed to match the level of significance) fit to the mean number of observations for any given type combination in the Monte Carlo runs. For example, if 28 = 256 counts are expected in the given permutation model, then roughly 340 observations in the real data are needed to satisfy p = 0.0001. If only 16 counts are expected, the needed number observed counts increases (as a ratio relative to expected) to about 40 for the same level of significance. Rarely observed type combinations with very rare expected values in the permutation models (i.e. candidates for over observed combinations, not under observed) were excluded from the analysis of significance. This is because, for example, it is difficult to determine the true significance or impact of a 4 type combination that is seen in the observed data only once, even if it was expected <0.001 times (for a very large observed/expected ratio) in a database of 30,000 specimens. (TIF) [file pone.0082761.s001.tif]

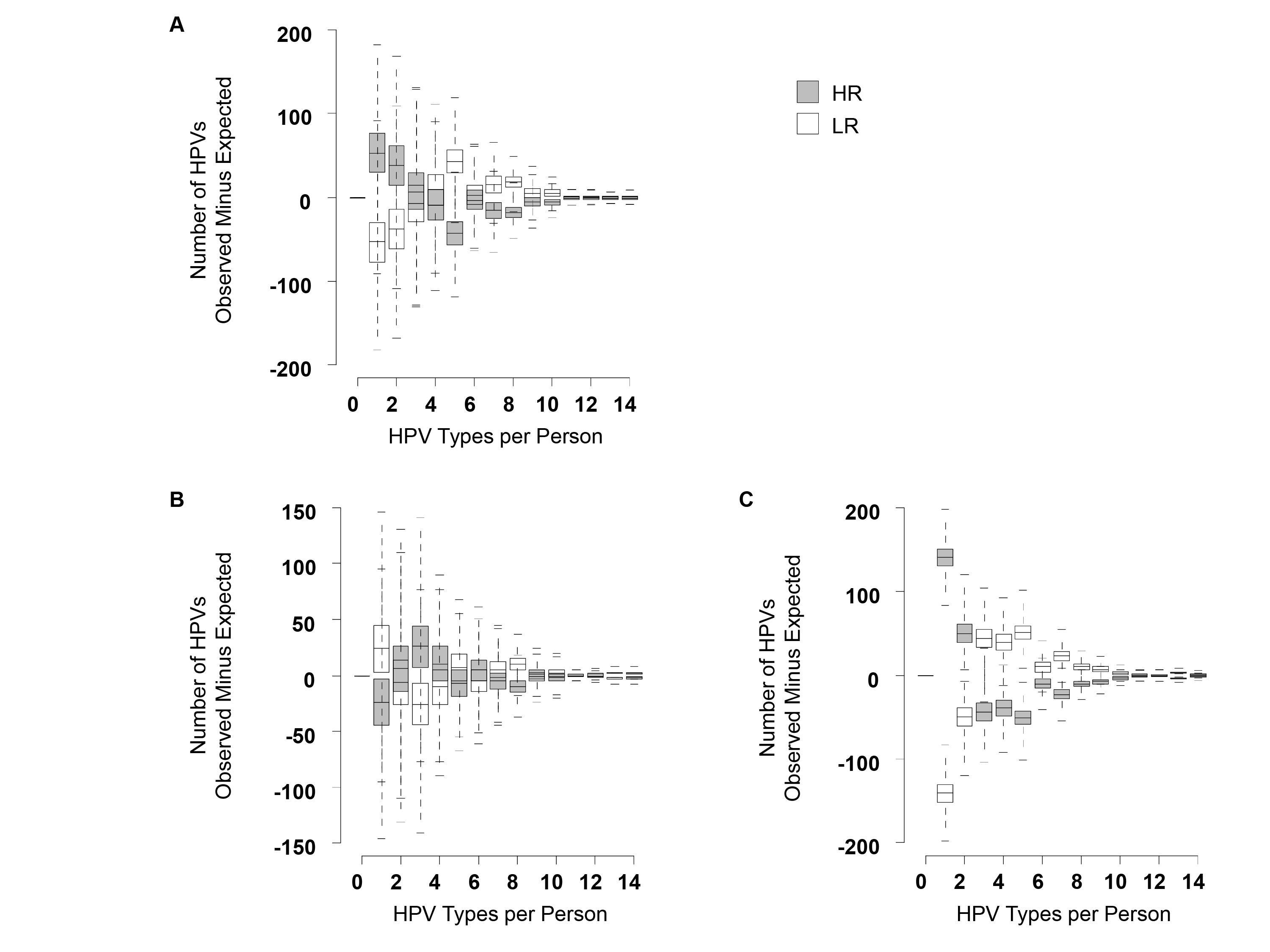

Supplement: Figure S2 — Difference between the observed data and the 10,000 non-strata model segregated by high-risk and low-risk HPV types: all subjects (A), general population (B), colposcopy population (C). (TIF) [file pone.0082761.s002.tif]

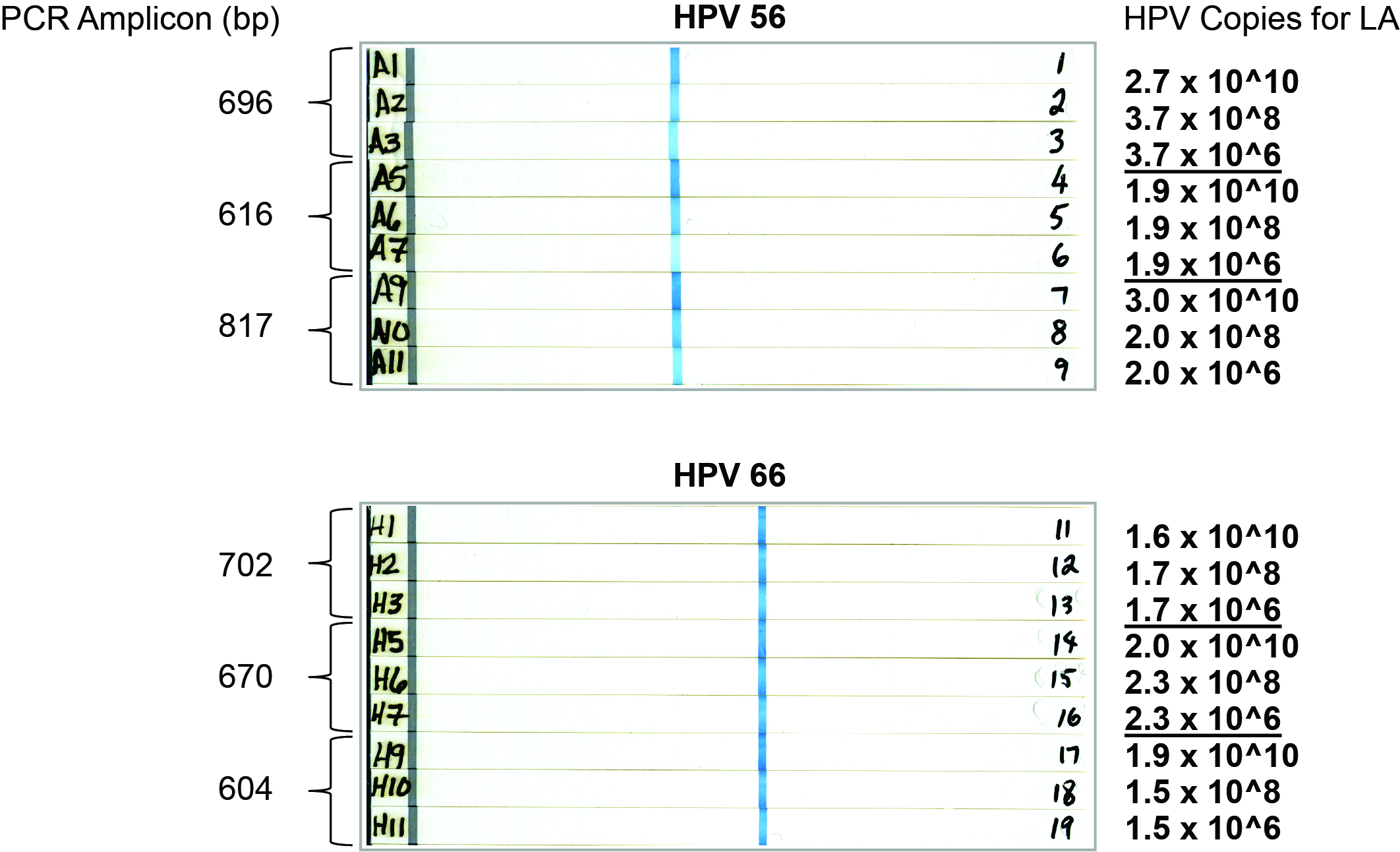

Supplement: Figure S3 — Testing specificity of HPV DNA genotyping test on PCR amplified plasmid DNA for (A) HPV 56 and (B) 66. (TIF) [file pone.0082761.s003.tif]
