# Supplementary material for: Randomization Modeling to Ascertain Clustering Patterns of Human Papillomavirus Types Detected in Cervicovaginal Samples in the United States
Source: PLoS One. 2013 Dec 18;8(12):e82761. doi: 10.1371/journal.pone.0082761 (PMC3867389; doi:10.1371/journal.pone.0082761)
Supplement: Table S1 — Primer sequences used for HPV 56 and 66 PCR amplification. (DOC) [file pone.0082761.s004.doc]

**Table S1. Primer sequences used for HPV 56 and 66 PCR amplification.**

| **HPV type** | **Direction** | **Prime Sequence** | **Position** | **Amplicon (bp)** |
| --- | --- | --- | --- | --- |
| 56 | forward | AATAGGGCTGGTAAAGTTGG | 6378 | 696 |
| 56 | reverse | TGTAGAGGTGGAGGTAGGAG | 7054 |
| 56 | forward | TTCTGTATATGTTGCTACGCC | 6481 | 616 |
| 56 | reverse | TGTAGAGGTGGAGGTAGGAG | 7054 |
| 56 | forward | CAGGGAACAATTATTTGCCAG | 6347 | 817 |
| 56 | reverse | AGCACATACACAAACATACC | 7144 |
| 66 | forward | TTTAATAGGGCAGGTAATGTTGGG | 6425 | 702 |
| 66 | reverse | TAAAGAAGAGGAAGAGGTAGGAG | 7126 |
| 66 | forward | CAGATTTGTATTGGAAGGGT | 6462 | 670 |
| 66 | reverse | GCTGGTAAAGAAGAGGAAGAG | 7111 |
| 66 | forward | TAGTGGGTCCATGATTACCT | 6529 | 604 |
| 66 | reverse | AGCTGGTAAAGAAGAGGAAGAG | 7111 |

HPV 56 and 66 plasmids were amplified each in 3 separate reactions before incorporation into the Linear Array assay. Two HPV56 reactions had a common reverse primer.
