# Supplementary material for: Randomization Modeling to Ascertain Clustering Patterns of Human Papillomavirus Types Detected in Cervicovaginal Samples in the United States
Source: PLoS One. 2013 Dec 18;8(12):e82761. doi: 10.1371/journal.pone.0082761 (PMC3867389; doi:10.1371/journal.pone.0082761)
Supplement: Table S2 — Key parameters and assumptions of the different randomization models. (DOC) [file pone.0082761.s005.doc]

**Table S2. Key parameters and assumptions of the different randomization models.**

|  |  | **Restricted Structure** | |  |
| --- | --- | --- | --- | --- |
| **Model** | **Randomization Function** | **Fixed Margins** | **Stratification** | **Criteria/Assumptions for Permutations** |
| **Null** | sample | Column | --- | - Frequency of each HPV type is preserved |
|  |  |  |  | - All subjects are from a homogeneous population with equal probabilities of acquiring HPV |
|  |  |  |  | - Factors such as disease status do not affect distribution of HPV types; all HPV types have equivalent distributions across strata of number of types per person |
|  |  |  |  | - Occurrences of HPV types in the different studies of the aggregate dataset are interchangeable |
|  |  |  |  | - Assortment of HPV type is independent of other types |
| **Non-Strata** | permatswap | Column | --- | - Frequency of each HPV type is preserved |
|  |  | Row |  | - *All subjects do not have equal probability of acquiring HPV; distribution of total number of HPV types per person in randomized data matches that of observed data* |
|  |  |  |  | - Factors such as disease status do not affect distribution of HPV types; all HPV types have equivalent distributions across strata of number of types per person |
|  |  |  |  | - Occurrences of HPV types in the different studies of the aggregate dataset are interchangeable |
|  |  |  |  | - Assortment of HPV type is independent of other types |
| **K Strata** | permatswap | Column |  | - Frequency of each HPV type is preserved |
|  |  | Row |  | - *All subjects do not have equal probability of acquiring HPV; distribution of number of HPV types per person in randomized data matches that of observed data* |
|  |  |  | Number of HPV types per person | - *All HPV types do not have equal distributions across number of HPV types per person; randomization of HPV type occurrence is restricted to the k strata in which it was observed* |
|  |  |  |  | - Occurrences of HPV types in the different studies of the aggregate dataset are interchangeable |
|  |  |  |  | - Assortment of HPV type is independent of other types *within the strata* |
| **Study Strata** | permatswap | Column |  | - Frequency of each HPV type is preserved |
|  |  | Row |  | - *All subjects do not have equal probability of acquiring HPV; distribution of number of HPV types per person in randomized data matches that of observed data* |
|  |  |  | Study | - Factors such as disease status do not affect distribution of HPV types; all HPV types have equivalent distributions across strata of number of types per person |
|  |  |  |  | - *Studies differ and HPV occurrences are not interchangeable; randomization of HPV type occurrence is restricted to the study strata in which it was observed* |
|  |  |  |  | - Assortment of HPV type is independent of other types *within the strata* |
| **Study-K Strata** | permatswap | Column |  | - Frequency of each HPV type is preserved |
|  |  | Row |  | - *All subjects do not have equal probability of acquiring HPV; distribution of number of HPV types per person in randomized data matches that of observed data* |
|  |  |  | Number of HPV types per person | - *All HPV types do not have equal distributions across number of HPV types per person; randomization of HPV type occurrence is restricted to the k strata in which it was observed* |
|  |  |  | Study | - *Studies differ and HPV occurrences are not interchangeable; randomization of HPV type occurrence is restricted to the study strata in which it was observed* |
|  |  |  |  | - Assortment of HPV type is independent of other types *within the strata* |
