# Supplementary material for: Randomization Modeling to Ascertain Clustering Patterns of Human Papillomavirus Types Detected in Cervicovaginal Samples in the United States
Source: PLoS One. 2013 Dec 18;8(12):e82761. doi: 10.1371/journal.pone.0082761 (PMC3867389; doi:10.1371/journal.pone.0082761)
Supplement: Table S3 — Significant 3 HPV type combinations. (DOC) [file pone.0082761.s006.doc]

**Table S3. Significant 3 HPV type combinations.**

|  |  |  | **Observed/Expected (Z-score) for Permutation Models** | | | |
| --- | --- | --- | --- | --- | --- | --- |
|  |  |  |  |  |  |  |
| **HPV Types*** | **Species** | **Observations** | **Non-Strata** | **Study Strata** | **k Strata** | **Study-k Strata** |
| **33,52,58** | α9, α9, α9 | 15 | 5.0 (6.9) | 3.6 (5.3) | 5.3 (7.2) | 5.7 (7.7) |
| **56,66,84** | α6, α6, α3 | 31 | 3.0 (6.4) | 3.0 (6.4) | 2.4 (5.0) | 2.8 (6.0) |
| **62,70,83** | α3, α7, α3 | 14 | 3.2 (4.6) | 2.5 (3.6) | 3.6 (5.1) | 2.9 (4.2) |
| **39,56,66** | α7, α6, α6 | 25 | 2.5 (4.8) | 2.5 (4.6) | 2.2 (4.0) | 2.2 (4.1) |
| **56,66,89** | α6, α6, α3 | 29 | 3.1 (6.4) | 3.0 (6.3) | 2.1 (4.2) | 2.1 (4.0) |
| **61,70,81** | α3, α7, α3 | 9 | 3.4 (3.9) |  | 4.3 (4.8) |  |
| **31,56,66** | α9, α6, α6 | 23 | 2.6 (4.7) | 2.3 (4.1) | 2.4 (4.3) |  |
| **40,45,68** | α8, α7, α7 | 7 | 5.8 (5.3) | 6.0 (5.4) | 4.3 (4.2) |  |
| **67,82,84** | α9, α5, α3 | 7 | 4.1 (4.1) | 4.4 (4.3) | 4.1 (4.1) |  |
| **61,62,83** | α3, α3, α3 | 22 | 2.4 (4.2) |  | 2.3 (4.1) |  |
| **56,59,66** | α6, α7, α6 | 26 | 2.7 (5.3) | 2.8 (5.4) | 2.1 (4.0) |  |
| **51,66,89** | α5, α6, α3 | 35 | 2.3 (5.0) | 2.2 (4.8) | 1.9 (4.0) |  |
| **61,70,89** | α3, α7, α3 | 13 | 2.6 (3.6) |  | 2.8 (3.9) |  |
| **16,39,82** | α9, α7, α5 | 15 |  |  | 2.6 (3.9) |  |
| **51,82,84** | α5, α5, α3 | 13 | 2.8 (3.8) | 2.9 (4.1) | 2.8 (3.8) |  |
| **62,81,83** | α3, α3, α3 | 14 | 2.7 (3.9) |  | 2.7 (3.8) |  |
| **61,68,83** | α3, α7, α3 | 13 | 2.8 (3.9) | 2.5 (3.3) | 2.7 (3.7) |  |
| **62,70,89** | α3, α7, α3 | 15 | 2.6 (3.8) |  | 2.5 (3.7) |  |
| **61,62,70** | α3, α3, α7 | 13 | 2.3 (3.1) |  | 2.7 (3.7) |  |
| **54,62,83** | α13, α3, α3 | 22 | 2.4 (4.3) | 2.1 (3.5) | 2.1 (3.6) |  |
| **16,18,39** | α9, α7, α7 | 30 |  |  | 1.9 (3.6) |  |
| **54,55,62** | α13, α10, α3 | 17 | 2.8 (4.5) | 2.5 (3.8) | 2.3 (3.5) |  |
| **16,45,54** | α9, α7, α13 | 23 |  |  | 2.0 (3.5) |  |
| **35,40,68** | α9, α8, α7 | 7 | 5.6 (5.2) | 5.7 (5.2) |  |  |
| **53,56,66** | α6, α6, α6 | 30 | 2.4 (5.0) | 2.4 (4.8) |  |  |
| **40,56,59** | α8, α6, α7 | 10 | 4.2 (4.9) | 4.1 (4.8) |  |  |
| **35,40,56** | α9, α8, α6 | 8 | 5.1 (5.1) | 4.3 (4.5) |  |  |
| **42,66,89** | α1, α6, α3 | 25 | 2.6 (4.9) | 2.4 (4.5) |  |  |
| **40,73,84** | α8, α11, α3 | 8 | 4.3 (4.5) | 4.2 (4.4) |  |  |
| **40,51,54** | α8, α5, α13 | 12 | 3.4 (4.5) | 3.3 (4.3) |  |  |
| **40,42,51** | α8, α1, α5 | 11 | 3.5 (4.5) | 3.3 (4.2) |  |  |
| **40,68,73** | α8, α7, α11 | 5 | 5.4 (4.2) | 5.4 (4.2) |  |  |
| **40,42,54** | α8, α1, α13 | 9 | 3.9 (4.4) | 3.6 (4.1) |  |  |
| **42,51,66** | α1, α5, α6 | 27 | 2.2 (4.2) | 2.1 (4.0) |  |  |
| **35,40,42** | α9, α8, α1 | 7 | 4.2 (4.1) | 4.0 (4.0) |  |  |
| **11,54,61** | α10, α13, α3 | 6 | 3.9 (3.6) | 4.4 (4.0) |  |  |
| **51,56,82** | α5, α6, α5 | 10 | 3.1 (3.8) | 3.2 (3.9) |  |  |
| **53,56,89** | α6, α6, α3 | 25 | 2.1 (3.9) | 2.1 (3.8) |  |  |
| **55,58,84** | α10, α9, α3 | 13 | 2.8 (3.8) | 2.8 (3.8) |  |  |
| **11,42,66** | α10, α1, α6 | 6 | 4.2 (3.8) | 4.2 (3.8) |  |  |
| **40,42,73** | α8, α1, α11 | 6 | 4.4 (3.9) | 4.1 (3.7) |  |  |
| **6,55,62** | α10, α10, α3 | 14 | 2.7 (3.9) | 2.6 (3.7) |  |  |
| **52,73,83** | α9, α11, α3 | 15 | 2.6 (3.8) | 2.5 (3.7) |  |  |
| **40,42,68** | α8, α1, α7 | 6 | 4.0 (3.7) | 4.0 (3.7) |  |  |
| **11,62,89** | α10, α3, α3 | 7 | 3.6 (3.6) | 3.6 (3.7) |  |  |
| **6,66,89** | α10, α6, α3 | 21 | 2.2 (3.6) | 2.2 (3.7) |  |  |
| **42,52,83** | α1, α9, α3 | 19 | 2.2 (3.4) | 2.3 (3.7) |  |  |
| **56,66,83** | α6, α6, α3 | 17 | 2.5 (3.9) | 2.4 (3.6) |  |  |
| **35,66,71** | α9, α6, α15 | 6 | 3.9 (3.6) | 3.9 (3.6) |  |  |
| **16,40,73** | α9, α8, α11 | 10 | 2.9 (3.6) | 3.0 (3.6) |  |  |
| **11,16,18** | α10, α9, α7 | 9 | 3.0 (3.5) | 3.1 (3.6) |  |  |
| **42,56,66** | α1, α6, α6 | 18 | 2.4 (3.9) | 2.2 (3.5) |  |  |
| **35,71,83** | α9, α15, α3 | 5 | 4.7 (3.8) | 4.3 (3.5) |  |  |
| **52,56,66** | α9, α6, α6 | 24 | 2.0 (3.5) | 2.0 (3.4) |  |  |
| **18,26,51** | α7, α5, α5 | 5 | 4.1 (3.4) | 4.1 (3.4) |  |  |
| **42,67,84** | α1, α9, α3 | 12 | 2.5 (3.3) | 2.6 (3.4) |  |  |
| **42,52,56** | α1, α9, α6 | 19 | 2.1 (3.3) | 2.1 (3.4) |  |  |
| **66,67,89** | α6, α9, α3 | 14 | 2.5 (3.6) | 2.3 (3.3) |  |  |
| **35,71,84** | α9, α15, α3 | 6 | 3.8 (3.5) | 3.6 (3.3) |  |  |
| **42,56,89** | α1, α6, α3 | 16 | 2.3 (3.4) | 2.2 (3.3) |  |  |
| **42,73,83** | α1, α11, α3 | 10 | 2.7 (3.3) | 2.7 (3.3) |  |  |
| **40,61,68** | α8, α3, α7 | 6 | 3.6 (3.3) | 3.5 (3.3) |  |  |
| **6,56,66** | α10, α6, α6 | 16 | 2.2 (3.3) | 2.2 (3.3) |  |  |
| **42,56,67** | α1, α6, α9 | 9 | 2.8 (3.2) | 2.9 (3.3) |  |  |
| **42,52,73** | α1, α9, α11 | 14 | 2.3 (3.1) | 2.4 (3.3) |  |  |
| **39,40,73** | α7, α8, α11 | 6 | 3.3 (3.1) | 3.6 (3.3) |  |  |
| **31,73,82** | α9, α11, α5 | 6 | 3.4 (3.1) | 3.6 (3.3) |  |  |
| **35,42,73** | α9, α1, α11 | 9 | 2.6 (3.0) | 2.8 (3.3) |  |  |
| **39,40,68** | α7, α8, α7 | 6 |  | 3.5 (3.3) |  |  |
| **40,51,84** | α8, α5, α3 | 11 | 2.6 (3.2) | 2.5 (3.2) |  |  |
| **6,51,89** | α10, α5, α3 | 22 | 2.0 (3.2) | 2.0 (3.2) |  |  |
| **51,66,82** | α5, α6, α5 | 11 | 2.5 (3.1) | 2.5 (3.2) |  |  |
| **35,55,62** | α9, α10, α3 | 11 | 2.5 (3.1) | 2.5 (3.2) |  |  |
| **35,39,40** | α9, α7, α8 | 7 | 3.1 (3.1) | 3.2 (3.2) |  |  |
| **11,18,53** | α10, α7, α6 | 6 | 3.2 (3.0) | 3.4 (3.2) |  |  |
| **6,33,54** | α10, α9, α13 | 7 |  | 3.2 (3.2) |  |  |
| **55,62,81** | α10, α3, α3 | 11 | 3.1 (3.9) | 2.5 (3.1) |  |  |
| **40,51,59** | α8, α5, α7 | 10 | 2.6 (3.1) | 2.6 (3.1) |  |  |
| **58,71,83** | α9, α15, α3 | 5 | 4.7 (3.8) |  |  |  |
| **55,62,83** | α10, α3, α3 | 13 | 2.7 (3.8) |  |  |  |
| **61,71,83** | α3, α15, α3 | 6 | 4.0 (3.7) |  |  |  |
| **55,81,83** | α10, α3, α3 | 8 | 3.4 (3.7) |  |  |  |
| **55,71,84** | α10, α15, α3 | 5 | 4.2 (3.5) |  |  |  |
| **61,72,83** | α3, α3, α3 | 7 | 3.3 (3.4) |  |  |  |
| **55,62,68** | α10, α3, α7 | 10 | 2.7 (3.3) |  |  |  |
| **54,61,81** | α13, α3, α3 | 13 | 2.4 (3.3) |  |  |  |
| **53,54,61** | α6, α13, α3 | 25 | 1.9 (3.3) |  |  |  |
| **40,56,81** | α8, α6, α3 | 5 | 3.9 (3.3) |  |  |  |
| **35,81,83** | α9, α3, α3 | 9 | 2.8 (3.3) |  |  |  |
| **33,61,81** | α9, α3, α3 | 6 | 3.5 (3.3) |  |  |  |
| **40,61,62** | α8, α3, α3 | 9 | 2.8 (3.2) |  |  |  |
| **40,55,62** | α8, α10, α3 | 6 | 3.5 (3.2) |  |  |  |
| **40,54,61** | α8, α13, α3 | 8 | 2.9 (3.2) |  |  |  |
| **6,61,89** | α10, α3, α3 | 18 | 2.1 (3.2) |  |  |  |
| **26,51,54** | α5, α5, α13 | 5 | 3.8 (3.2) |  |  |  |
| **55,73,83** | α10, α11, α3 | 7 | 3.0 (3.1) |  |  |  |
| **40,42,84** | α8, α1, α3 | 8 | 2.8 (3.1) |  |  |  |
| **58,68,83** | α9, α7, α3 | 9 | 2.6 (3.0) |  |  |  |
| **35,61,62** | α9, α3, α3 | 17 | 2.0 (3.0) |  |  |  |
| **16,56,66** | α9, α6, α6 | 33 | 1.7 (3.0) |  |  |  |
| **51,67,82** | α5, α9, α5 | 6 | 3.2 (3.0) |  |  |  |
| **18,51,82** | α7, α5, α5 | 9 | 2.6 (3.0) |  |  |  |
| **6,11,66** | α10, α10, α6 | 5 | 3.6 (3) |  |  |  |
| **81,83,84** | α3, α3, α3 | 12 | 2.3 (2.9) |  |  |  |
| **53,66,89** | α6, α6, α3 | 28 | 1.7 (2.9) |  |  |  |
| **18,56,66** | α7, α6, α6 | 16 | 2.1 (2.9) |  |  |  |
| **62,67,84** | α3, α9, α3 | 14 | 2.1 (2.9) |  |  |  |
| **53,55,62** | α6, α10, α3 | 17 | 2.0 (2.9) |  |  |  |
| **51,66,73** | α5, α6, α11 | 16 | 2.0 (2.9) |  |  |  |
| **42,53,56** | α1, α6, α6 | 18 | 1.9 (2.9) |  |  |  |
| **39,40,66** | α7, α8, α6 | 9 | 2.6 (2.9) |  |  |  |
| **39,40,56** | α7, α8, α6 | 7 | 2.9 (2.9) |  |  |  |
| **31,51,82** | α9, α5, α5 | 10 | 2.4 (2.9) |  |  |  |
| **6,52,73** | α10, α9, α11 | 13 | 2.2 (2.9) |  |  |  |

Results are listed for HPV combinations with fdr ≤ 0.05. All 3-way associations were observed more than expected; no under observed 3-way combinations were significant. *HR-HPV types are underlined.
