# Supplementary material for: Randomization Modeling to Ascertain Clustering Patterns of Human Papillomavirus Types Detected in Cervicovaginal Samples in the United States
Source: PLoS One. 2013 Dec 18;8(12):e82761. doi: 10.1371/journal.pone.0082761 (PMC3867389; doi:10.1371/journal.pone.0082761)
Supplement: Table S4 — Significant 4 HPV type combinations. (DOC) [file pone.0082761.s007.doc]

**Table S4. Significant 4 HPV type combinations.**

|  |  |  | **Observed/Expected (Z-score) for Permutation Models** | | | |
| --- | --- | --- | --- | --- | --- | --- |
|  |  |  |  | | | |
| **HPV Types*** | **Species** | **Observations** | **Non-Strata** | **Study Strata** | **k Strata** | **Study-k Strata** |
| **31,56,66,84** | α9, α6, α6, α3 | 8 | 4.8 (4.9) | 4.7 (4.8) | 5.1 (5.1) | 6.0 (5.8) |
| **16,31,39,82** | α9, α9, α7, α5 | 5 | 4.1 (3.4) | 4.1 (3.4) | 8.5 (5.7) | 8.3 (5.7) |
| **42,56,66,83** | α1, α6, α6, α3 | 7 | 7.4 (6.2) | 7.4 (6.2) | 4.7 (4.5) | 5.9 (5.4) |
| **31,56,66,89** | α9, α6, α6, α3 | 9 | 6.0 (6.1) | 5.6 (5.8) | 5.1 (5.4) | 4.8 (5.2) |
| **6,18,61,89** | α10, α7, α3, α3 | 6 | 4.9 (4.3) | 5.1 (4.4) | 4.7 (4.2) | 6.4 (5.2) |
| **16,35,39,61** | α9, α9, α7, α3 | 7 | 2.7 (2.8) | 2.9 (2.9) | 3.8 (3.8) | 5.4 (5.0) |
| **42,51,58,81** | α1, α5, α9, α3 | 5 | 6.3 (4.7) | 6.9 (5.0) | 7.4 (5.3) | 6.5 (4.8) |
| **42,51,66,89** | α1, α5, α6, α3 | 12 | 6.0 (7.1) | 5.8 (6.9) | 4.3 (5.6) | 3.5 (4.6) |
| **16,53,58,89** | α9, α6, α9, α3 | 8 | 2.2 (2.3) | 2.4 (2.5) | 3.5 (3.8) | 4.1 (4.4) |
| **6,31,66,89** | α10, α9, α6, α3 | 7 | 4.4 (4.3) | 4.9 (4.6) | 5.1 (4.8) | 4.3 (4.2) |
| **31,39,56,66** | α9, α7, α6, α6 | 6 | 3.8 (3.5) | 3.5 (3.2) | 4.5 (4.0) | 4.7 (4.2) |
| **6,53,56,66** | α10, α6, α6, α6 | 8 | 4.9 (5.0) | 5.1 (5.1) | 3.6 (3.9) | 3.9 (4.2) |
| **61,62,70,89** | α3, α3, α7, α3 | 6 | 6.3 (5.2) | 4.0 (3.7) | 7.1 (5.6) | 4.6 (4.1) |
| **6,39,53,59** | α10, α7, α6, α7 | 6 | 2.8 (2.6) | 3.2 (3.0) | 3.5 (3.3) | 4.2 (3.9) |
| **54,55,62,66** | α13, α10, α3, α6 | 5 | 4.8 (3.9) | 4.2 (3.5) | 3.4 (2.9) | 4.8 (3.9) |
| **51,53,56,67** | α5, α6, α6, α9 | 6 | 5.3 (4.6) | 5.9 (5.0) | 5.7 (4.8) | 4.2 (3.8) |
| **35,42,73,83** | α9, α1, α11, α3 | 5 | 10.8 (6.7) | 10.8 (6.7) | 5.8 (4.4) | 4.7 (3.8) |
| **52,53,55,62** | α9, α6, α10, α3 | 8 | 4.6 (4.7) | 4.2 (4.4) | 3.0 (3.3) | 3.6 (3.8) |
| **35,52,61,83** | α9, α9, α3, α3 | 6 | 5.3 (4.6) | 4.6 (4.1) | 4.5 (4.1) | 4.0 (3.7) |
| **42,56,66,89** | α1, α6, α6, α3 | 8 | 6.7 (6.2) | 6.0 (5.8) | 3.4 (3.6) | 3.4 (3.7) |
| **31,56,84,89** | α9, α6, α3, α3 | 5 | 3.1 (2.6) | 3.2 (2.8) | 3.2 (2.8) | 4.5 (3.7) |
| **16,18,59,81** | α9, α7, α7, α3 | 5 | 2.8 (2.4) | 2.9 (2.5) | 5.3 (4.2) | 4.4 (3.6) |
| **56,66,84,89** | α6, α6, α3, α3 | 7 | 3.9 (3.9) | 4.1 (4.0) | 3.0 (3.1) | 3.6 (3.6) |
| **31,52,56,66** | α9, α9, α6, α6 | 9 | 4.8 (5.2) | 4.2 (4.7) | 4.3 (4.8) | 3.0 (3.5) |
| **53,56,66,89** | α6, α6, α6, α3 | 10 | 4.9 (5.6) | 4.7 (5.4) | 3.1 (3.8) | 2.9 (3.5) |
| **16,42,45,54** | α9, α1, α7, α13 | 5 | 2.5 (2.1) | 2.6 (2.2) | 3.4 (2.9) | 4.2 (3.5) |
| **56,59,66,84** | α6, α7, α6, α3 | 6 | 3.4 (3.2) | 3.6 (3.4) | 3.0 (2.8) | 3.7 (3.5) |
| **35,39,52,61** | α9, α7, α9, α3 | 5 | 3.0 (2.6) | 3.1 (2.7) | 2.6 (2.2) | 4.2 (3.5) |
| **51,56,66,89** | α5, α6, α6, α3 | 9 | 4.7 (5.1) | 4.6 (5.0) | 3.4 (3.9) | 3.0 (3.4) |
| **39,56,59,66** | α7, α6, α7, α6 | 6 | 3.6 (3.3) | 3.7 (3.4) | 3.6 (3.4) | 3.6 (3.4) |
| **56,62,66,84** | α6, α3, α6, α3 | 7 | 3.7 (3.7) | 3.7 (3.7) | 3.0 (3.0) | 3.3 (3.4) |
| **31,61,84,89** | α9, α3, α3, α3 | 5 | 2.5 (2.1) | 2.4 (2.0) | 3.5 (3.0) | 4.1 (3.4) |
| **6,54,55,62** | α10, α13, α10, α3 | 6 | 7.0 (5.6) | 6.4 (5.2) | 5.8 (4.9) | 3.4 (3.2) |
| **53,54,59,66** | α6, α13, α7, α6 | 9 | 3.6 (4.2) | 3.7 (4.2) | 3.4 (4.0) | 2.8 (3.2) |
| **61,62,83,84** | α3, α3, α3, α3 | 6 | 3.6 (3.4) | 2.4 (2.2) | 3.5 (3.3) | 3.4 (3.2) |
| **31,56,61,66** | α9, α6, α3, α6 | 5 | 3.6 (3.1) | 3.1 (2.7) | 3.4 (2.9) | 3.7 (3.2) |
| **16,35,61,62** | α9, α9, α3, α3 | 6 |  | 2.2 (2.0) | 2.9 (2.7) | 3.4 (3.2) |
| **16,35,59,62** | α9, α9, α7, α3 | 6 |  | 2.4 (2.2) | 2.8 (2.6) | 3.4 (3.2) |
| **6,55,62,66** | α10, α10, α3, α6 | 5 | 5.4 (4.2) | 5.4 (4.2) | 4.6 (3.8) | 3.6 (3.1) |
| **31,53,66,89** | α9, α6, α6, α3 | 8 | 3.1 (3.4) | 3.1 (3.4) | 3.4 (3.7) | 2.8 (3.1) |
| **6,51,66,73** | α10, α5, α6, α11 | 5 | 4.7 (3.8) | 5.3 (4.2) | 4.1 (3.4) | 3.7 (3.1) |
| **6,53,62,66** | α10, α6, α3, α6 | 7 | 2.9 (3.0) | 3.0 (3.0) | 2.9 (3.0) | 3.0 (3.1) |
| **53,54,59,68** | α6, α13, α7, α7 | 6 | 4.4 (4.0) | 4.3 (3.9) | 3.0 (2.8) | 3.3 (3.1) |
| **31,42,56,89** | α9, α1, α6, α3 | 5 | 4.4 (3.6) | 4.4 (3.6) | 3.2 (2.7) | 3.6 (3.1) |
| **31,51,56,84** | α9, α5, α6, α3 | 5 | 2.6 (2.2) | 2.6 (2.2) | 3.0 (2.6) | 3.7 (3.1) |
| **16,39,56,66** | α9, α7, α6, α6 | 8 | 2.5 (2.6) | 2.3 (2.4) | 3.1 (3.4) | 2.8 (3.0) |
| **31,66,84,89** | α9, α6, α3, α3 | 6 | 2.8 (2.6) | 2.8 (2.6) | 3.2 (3.1) | 3.1 (3.0) |
| **42,52,73,89** | α1, α9, α11, α3 | 5 | 4.7 (3.8) | 5.3 (4.2) | 2.6 (2.3) | 3.5 (3.0) |
| **54,62,83,84** | α13, α3, α3, α3 | 5 | 3.1 (2.7) |  |  | 3.5 (3.0) |
| **45,53,59,89** | α7, α6, α7, α3 | 6 | 3.5 (3.3) | 3.9 (3.6) | 3.5 (3.3) | 3.1 (2.9) |
| **53,56,59,89** | α6, α6, α7, α3 | 8 | 4.1 (4.4) | 4.3 (4.5) | 2.9 (3.2) | 2.7 (2.9) |
| **6,18,62,89** | α10, α7, α3, α3 | 5 | 3.6 (3.0) | 3.5 (3.0) | 3.0 (2.6) | 3.3 (2.9) |
| **42,53,56,66** | α1, α6, α6, α6 | 7 | 4.3 (4.2) | 3.9 (3.9) | 2.5 (2.5) | 2.9 (2.9) |
| **31,53,56,66** | α9, α6, α6, α6 | 8 | 4.1 (4.4) | 3.7 (4.0) | 3.5 (3.8) | 2.6 (2.8) |
| **31,53,56,89** | α9, α6, α6, α3 | 6 | 3.3 (3.1) | 3.2 (3.0) | 2.7 (2.6) | 3.0 (2.8) |
| **53,55,59,62** | α6, α10, α7, α3 | 5 | 3.5 (3.0) | 3.3 (2.8) | 2.7 (2.3) | 3.2 (2.8) |
| **16,52,59,89** | α9, α9, α7, α3 | 8 |  |  | 2.2 (2.3) | 2.6 (2.8) |
| **16,52,59,61** | α9, α9, α7, α3 | 7 |  |  | 2.2 (2.2) | 2.7 (2.8) |
| **55,58,83,84** | α10, α9, α3, α3 | 5 | 8.4 (5.7) | 7.6 (5.4) | 7.3 (5.2) | 3.2 (2.7) |
| **54,61,68,83** | α13, α3, α7, α3 | 5 | 6.8 (5.0) | 5.2 (4.1) | 3.3 (2.8) | 3.1 (2.7) |
| **58,66,84,89** | α9, α6, α3, α3 | 5 | 3.1 (2.7) | 3.3 (2.8) | 3.3 (2.8) | 3.1 (2.7) |
| **16,52,54,68** | α9, α9, α13, α7 | 6 | 2.4 (2.2) | 2.5 (2.3) |  | 2.9 (2.7) |
| **31,39,51,54** | α9, α7, α5, α13 | 8 | 3.5 (3.8) | 3.6 (3.9) | 4.2 (4.5) | 2.5 (2.6) |
| **18,51,66,84** | α7, α5, α6, α3 | 7 | 3.1 (3.2) | 3.2 (3.2) | 3.6 (3.7) | 2.6 (2.6) |
| **16,18,39,73** | α9, α7, α7, α11 | 7 | 3.8 (3.8) | 4.2 (4.1) | 4.2 (4.1) |  |
| **53,54,61,84** | α6, α13, α3, α3 | 9 | 3.7 (4.2) | 3.0 (3.4) | 3.5 (4.1) |  |
| **51,53,56,89** | α5, α6, α6, α3 | 10 | 4.3 (5.0) | 4.2 (4.9) | 3.2 (3.9) |  |
| **56,58,61,84** | α6, α9, α3, α3 | 5 | 4.3 (3.5) | 4.3 (3.6) | 4.7 (3.8) |  |
| **16,33,53,84** | α9, α9, α6, α3 | 6 | 3.7 (3.5) | 3.9 (3.6) | 4.2 (3.8) |  |
| **16,35,39,45** | α9, α9, α7, α7 | 5 | 2.6 (2.2) | 2.5 (2.1) | 4.7 (3.8) |  |
| **6,51,66,84** | α10, α5, α6, α3 | 7 | 3.2 (3.3) | 3.4 (3.4) | 3.7 (3.7) |  |
| **39,52,55,89** | α7, α9, α10, α3 | 5 | 3.8 (3.2) | 4.1 (3.4) | 4.5 (3.7) |  |
| **16,52,54,58** | α9, α9, α13, α9 | 9 | 2.7 (3.1) | 2.6 (3.0) | 3.1 (3.6) |  |
| **31,51,66,84** | α9, α5, α6, α3 | 7 | 2.7 (2.7) | 2.7 (2.7) | 3.5 (3.5) |  |
| **54,58,61,83** | α13, α9, α3, α3 | 5 | 5.4 (4.3) | 4.7 (3.8) | 4.1 (3.4) |  |
| **6,51,66,89** | α10, α5, α6, α3 | 7 | 3.5 (3.5) | 3.7 (3.7) | 3.2 (3.3) |  |
| **16,59,68,73** | α9, α7, α7, α11 | 5 | 4.0 (3.4) | 4.4 (3.7) | 4.0 (3.3) |  |
| **42,53,62,70** | α1, α6, α3, α7 | 5 | 4.6 (3.7) | 4.2 (3.5) | 3.9 (3.3) |  |
| **16,54,58,83** | α9, α13, α9, α3 | 7 | 3.5 (3.6) | 3.2 (3.3) | 3.1 (3.2) |  |
| **51,56,58,61** | α5, α6, α9, α3 | 5 | 3.9 (3.3) | 3.9 (3.3) | 3.8 (3.2) |  |
| **16,39,59,73** | α9, α7, α7, α11 | 6 | 2.6 (2.4) | 3.0 (2.8) | 3.4 (3.2) |  |
| **51,61,62,68** | α5, α3, α3, α7 | 5 | 3.5 (3.0) | 3.0 (2.6) | 3.8 (3.2) |  |
| **16,18,39,42** | α9, α7, α7, α1 | 7 | 2.5 (2.5) | 2.5 (2.5) | 3.2 (3.2) |  |
| **18,31,39,52** | α7, α9, α7, α9 | 5 | 2.4 (2.0) | 2.5 (2.1) | 3.7 (3.2) |  |
| **16,31,56,66** | α9, α9, α6, α6 | 7 | 2.3 (2.3) |  | 3.1 (3.2) |  |
| **16,18,39,52** | α9, α7, α7, α9 | 8 |  |  | 3.0 (3.2) |  |
| **6,56,66,89** | α10, α6, α6, α3 | 6 | 4.9 (4.3) | 5.2 (4.5) | 3.3 (3.1) |  |
| **16,18,52,73** | α9, α7, α9, α11 | 7 | 3.3 (3.3) | 3.3 (3.4) | 3.1 (3.1) |  |
| **52,54,58,89** | α9, α13, α9, α3 | 5 | 3.0 (2.6) | 3.5 (3.0) | 3.6 (3.1) |  |
| **31,39,53,59** | α9, α7, α6, α7 | 6 | 2.3 (2.1) | 2.5 (2.3) | 3.3 (3.1) |  |
| **52,59,73,83** | α9, α7, α11, α3 | 5 | 4.9 (3.9) | 4.9 (3.9) | 3.5 (3.0) |  |
| **6,35,53,61** | α10, α9, α6, α3 | 5 | 3.9 (3.3) | 4.4 (3.6) | 3.5 (3.0) |  |
| **52,54,59,66** | α9, α13, α7, α6 | 7 | 2.9 (2.9) | 3.1 (3.2) | 2.9 (3.0) |  |
| **51,56,58,84** | α5, α6, α9, α3 | 5 | 3.5 (3.0) | 3.6 (3.1) | 3.5 (3.0) |  |
| **16,35,56,83** | α9, α9, α6, α3 | 5 | 3.1 (2.7) | 2.8 (2.4) | 3.5 (3.0) |  |
| **40,53,56,59** | α8, α6, α6, α7 | 5 | 9.7 (6.2) | 9.7 (6.2) | 3.4 (2.9) |  |
| **42,52,73,83** | α1, α9, α11, α3 | 5 | 6.1 (4.6) | 6.1 (4.6) | 3.4 (2.9) |  |
| **42,53,62,83** | α1, α6, α3, α3 | 7 | 4.2 (4.1) | 3.8 (3.8) | 2.8 (2.9) |  |
| **18,39,42,59** | α7, α7, α1, α7 | 5 | 3.5 (3.0) | 3.8 (3.2) | 3.4 (2.9) |  |
| **6,51,53,89** | α10, α5, α6, α3 | 7 | 3.0 (3.0) | 2.9 (3.0) | 2.9 (2.9) |  |
| **16,31,39,73** | α9, α9, α7, α11 | 5 | 2.3 (2) | 2.5 (2.1) | 3.4 (2.9) |  |
| **16,18,51,66** | α9, α7, α5, α6 | 8 |  |  | 2.7 (2.9) |  |
| **52,56,59,66** | α9, α6, α7, α6 | 7 | 3.6 (3.6) | 3.7 (3.7) | 2.8 (2.8) |  |
| **6,42,51,66** | α10, α1, α5, α6 | 6 | 3.7 (3.5) | 4.0 (3.7) | 3.0 (2.8) |  |
| **39,42,52,83** | α7, α1, α9, α3 | 5 | 3.2 (2.7) | 3.6 (3.1) | 3.2 (2.8) |  |
| **6,61,66,89** | α10, α3, α6, α3 | 5 | 3.2 (2.8) | 3.2 (2.7) | 3.3 (2.8) |  |
| **16,39,42,89** | α9, α7, α1, α3 | 7 | 2.1 (2.0) | 2.2 (2.2) | 2.7 (2.8) |  |
| **16,18,31,52** | α9, α7, α9, α9 | 7 |  |  | 2.7 (2.8) |  |
| **42,51,54,62** | α1, α5, α13, α3 | 7 | 3.5 (3.5) | 3.5 (3.5) | 2.7 (2.7) |  |
| **39,56,66,89** | α7, α6, α6, α3 | 6 | 3.6 (3.3) | 3.7 (3.4) | 2.9 (2.7) |  |
| **16,40,51,59** | α9, α8, α5, α7 | 5 | 3.6 (3.1) | 3.8 (3.2) | 3.1 (2.7) |  |
| **52,53,54,59** | α9, α6, α13, α7 | 8 | 2.7 (3.0) | 2.9 (3.1) | 2.5 (2.7) |  |
| **31,42,56,66** | α9, α1, α6, α6 | 5 | 4.5 (3.7) | 3.5 (3.0) | 3.2 (2.7) |  |
| **52,54,56,58** | α9, α13, α6, α9 | 5 | 4.0 (3.4) | 3.6 (3.0) | 3.1 (2.7) |  |
| **52,53,59,83** | α9, α6, α7, α3 | 7 | 3.0 (3.1) | 2.9 (3.0) | 2.7 (2.7) |  |
| **31,51,52,73** | α9, α5, α9, α11 | 5 | 3.2 (2.8) | 3.5 (3.0) | 3.2 (2.7) |  |
| **18,54,59,66** | α7, α13, α7, α6 | 5 | 3.0 (2.6) | 3.2 (2.8) | 3.2 (2.7) |  |
| **31,51,56,66** | α9, α5, α6, α6 | 6 | 3.3 (3.1) | 2.9 (2.7) | 2.9 (2.7) |  |
| **39,42,51,66** | α7, α1, α5, α6 | 6 | 2.7 (2.6) | 2.7 (2.6) | 2.8 (2.7) |  |
| **31,51,59,66** | α9, α5, α7, α6 | 6 | 2.4 (2.2) | 2.3 (2.1) | 2.9 (2.7) |  |
| **31,61,62,84** | α9, α3, α3, α3 | 5 |  |  | 3.2 (2.7) |  |
| **16,31,39,51** | α9, α9, α7, α5 | 7 |  |  | 2.7 (2.7) |  |
| **16,31,52,59** | α9, α9, α9, α7 | 7 |  |  | 2.7 (2.7) |  |
| **52,59,62,73** | α9, α7, α3, α11 | 6 | 4.0 (3.6) | 4.5 (4.1) | 2.8 (2.6) |  |
| **52,53,59,73** | α9, α6, α7, α11 | 6 | 3.5 (3.3) | 3.8 (3.5) | 2.7 (2.6) |  |
| **52,53,55,89** | α9, α6, α10, α3 | 6 | 3.9 (3.6) | 3.7 (3.4) | 2.8 (2.6) |  |
| **52,54,58,83** | α9, α13, α9, α3 | 5 | 4.2 (3.5) | 3.6 (3.1) | 3.0 (2.6) |  |
| **35,52,61,62** | α9, α9, α3, α3 | 6 | 3.3 (3.1) | 3.3 (3.1) | 2.7 (2.6) |  |
| **16,35,39,81** | α9, α9, α7, α3 | 5 | 3.3 (2.8) | 3.4 (2.9) | 3.1 (2.6) |  |
| **52,56,58,84** | α9, α6, α9, α3 | 5 | 3.4 (2.9) | 3.2 (2.8) | 3.0 (2.6) |  |
| **51,59,66,89** | α5, α7, α6, α3 | 7 | 2.7 (2.8) | 2.8 (2.8) | 2.6 (2.6) |  |
| **31,51,66,89** | α9, α5, α6, α3 | 6 | 2.5 (2.3) | 2.5 (2.3) | 2.8 (2.6) |  |
| **16,35,39,42** | α9, α9, α7, α1 | 5 |  |  | 3.0 (2.6) |  |
| **16,51,53,89** | α9, α5, α6, α3 | 10 |  |  | 2.2 (2.6) |  |
| **16,31,35,39** | α9, α9, α9, α7 | 5 |  |  | 3.0 (2.6) |  |
| **16,52,54,59** | α9, α9, α13, α7 | 8 |  |  | 2.5 (2.6) |  |
| **16,31,39,52** | α9, α9, α7, α9 | 7 |  |  | 2.6 (2.6) |  |
| **16,39,42,52** | α9, α7, α1, α9 | 7 |  |  | 2.6 (2.6) |  |
| **42,53,56,89** | α1, α6, α6, α3 | 7 | 4.8 (4.6) | 4.3 (4.2) | 2.5 (2.5) |  |
| **51,53,54,73** | α5, α6, α13, α11 | 6 | 4.1 (3.7) | 3.8 (3.5) | 2.7 (2.5) |  |
| **53,56,59,66** | α6, α6, α7, α6 | 7 | 3.3 (3.3) | 3.4 (3.5) | 2.5 (2.5) |  |
| **51,53,56,66** | α5, α6, α6, α6 | 8 | 3.1 (3.4) | 3.0 (3.3) | 2.3 (2.5) |  |
| **31,51,54,62** | α9, α5, α13, α3 | 6 | 2.6 (2.4) | 2.6 (2.4) | 2.6 (2.5) |  |
| **16,52,59,73** | α9, α9, α7, α11 | 6 | 2.3 (2.1) | 2.4 (2.2) | 2.7 (2.5) |  |
| **39,51,59,62** | α7, α5, α7, α3 | 6 |  | 2.4 (2.2) | 2.7 (2.5) |  |
| **6,16,53,89** | α10, α9, α6, α3 | 7 |  |  | 2.5 (2.5) |  |
| **16,45,52,54** | α9, α7, α9, α13 | 5 |  |  | 2.9 (2.5) |  |
| **16,39,52,58** | α9, α7, α9, α9 | 6 |  |  | 2.7 (2.5) |  |
| **52,56,59,73** | α9, α6, α7, α11 | 5 | 4.8 (3.9) | 5.3 (4.2) | 2.8 (2.4) |  |
| **42,51,53,56** | α1, α5, α6, α6 | 7 | 3.7 (3.7) | 3.6 (3.7) | 2.4 (2.4) |  |
| **42,59,66,89** | α1, α7, α6, α3 | 6 | 3.5 (3.3) | 3.6 (3.4) | 2.6 (2.4) |  |
| **18,53,58,61** | α7, α6, α9, α3 | 5 | 3.2 (2.8) | 3.7 (3.2) | 2.7 (2.4) |  |
| **51,52,66,89** | α5, α9, α6, α3 | 8 | 2.5 (2.7) | 2.6 (2.9) | 2.3 (2.4) |  |
| **52,53,62,89** | α9, α6, α3, α3 | 9 | 2.5 (2.9) | 2.5 (2.8) | 2.2 (2.4) |  |
| **31,42,66,89** | α9, α1, α6, α3 | 5 | 3.2 (2.7) | 3.2 (2.7) | 2.8 (2.4) |  |
| **6,66,84,89** | α10, α6, α3, α3 | 5 | 2.9 (2.5) | 2.8 (2.4) | 2.8 (2.4) |  |
| **16,42,53,89** | α9, α1, α6, α3 | 8 | 2.0 (2.0) | 2.0 (2.1) | 2.2 (2.4) |  |
| **31,51,54,59** | α9, α5, α13, α7 | 5 |  | 2.4 (2.0) | 2.8 (2.4) |  |
| **16,18,56,66** | α9, α7, α6, α6 | 6 | 2.3 (2.1) |  | 2.6 (2.4) |  |
| **51,61,62,84** | α5, α3, α3, α3 | 6 |  |  | 2.5 (2.4) |  |
| **31,51,84,89** | α9, α5, α3, α3 | 5 |  |  | 2.7 (2.4) |  |
| **16,39,42,59** | α9, α7, α1, α7 | 6 |  |  | 2.5 (2.4) |  |
| **16,42,73,83** | α9, α1, α11, α3 | 5 | 3.9 (3.3) | 4.0 (3.4) | 2.6 (2.3) |  |
| **6,42,66,89** | α10, α1, α6, α3 | 5 | 3.8 (3.2) | 3.9 (3.3) | 2.7 (2.3) |  |
| **51,66,73,89** | α5, α6, α11, α3 | 5 | 3.6 (3.1) | 3.9 (3.3) | 2.7 (2.3) |  |
| **6,53,56,59** | α10, α6, α6, α7 | 5 | 3.2 (2.8) | 3.6 (3.1) | 2.7 (2.3) |  |
| **53,56,58,89** | α6, α6, α9, α3 | 5 | 3.7 (3.2) | 3.5 (3.0) | 2.7 (2.3) |  |
| **42,51,53,83** | α1, α5, α6, α3 | 6 | 3.3 (3.1) | 3.2 (3.0) | 2.5 (2.3) |  |
| **42,51,66,83** | α1, α5, α6, α3 | 5 | 3.2 (2.8) | 3.4 (2.9) | 2.7 (2.3) |  |
| **42,51,53,89** | α1, α5, α6, α3 | 7 | 2.9 (3.0) | 2.7 (2.7) | 2.3 (2.3) |  |
| **31,51,53,54** | α9, α5, α6, α13 | 7 | 2.6 (2.6) | 2.5 (2.6) | 2.3 (2.3) |  |
| **42,51,66,84** | α1, α5, α6, α3 | 6 | 2.7 (2.5) | 2.7 (2.6) | 2.4 (2.3) |  |
| **31,42,51,54** | α9, α1, α5, α13 | 5 | 3.0 (2.6) | 2.9 (2.5) | 2.7 (2.3) |  |
| **39,42,51,54** | α7, α1, α5, α13 | 5 | 2.6 (2.2) | 2.7 (2.3) | 2.7 (2.3) |  |
| **6,53,61,89** | α10, α6, α3, α3 | 5 | 2.7 (2.3) | 2.4 (2.0) | 2.7 (2.3) |  |
| **6,51,84,89** | α10, α5, α3, α3 | 5 | 2.4 (2.0) | 2.4 (2.0) | 2.7 (2.3) |  |
| **51,53,58,89** | α5, α6, α9, α3 | 5 | 2.3 (2.0) |  | 2.7 (2.3) |  |
| **16,53,66,89** | α9, α6, α6, α3 | 9 |  |  | 2.1 (2.3) |  |
| **16,31,52,73** | α9, α9, α9, α11 | 5 |  |  | 2.7 (2.3) |  |
| **52,53,73,83** | α9, α6, α11, α3 | 5 | 3.8 (3.2) | 4.0 (3.4) | 2.6 (2.2) |  |
| **16,18,42,73** | α9, α7, α1, α11 | 5 | 3.6 (3.1) | 3.9 (3.3) | 2.6 (2.2) |  |
| **45,53,56,89** | α7, α6, α6, α3 | 5 | 3.8 (3.2) | 3.7 (3.1) | 2.6 (2.2) |  |
| **6,51,56,89** | α10, α5, α6, α3 | 5 | 3.5 (3.0) | 3.7 (3.1) | 2.6 (2.2) |  |
| **51,53,56,59** | α5, α6, α6, α7 | 7 | 2.9 (3.0) | 3.0 (3.0) | 2.2 (2.2) |  |
| **6,51,56,66** | α10, α5, α6, α6 | 5 | 3.2 (2.7) | 3.3 (2.8) | 2.6 (2.2) |  |
| **31,42,52,89** | α9, α1, α9, α3 | 5 | 2.6 (2.2) | 2.9 (2.5) | 2.6 (2.2) |  |
| **16,53,56,89** | α9, α6, α6, α3 | 8 | 2.1 (2.1) | 2.2 (2.3) | 2.1 (2.2) |  |
| **18,52,54,66** | α7, α9, α13, α6 | 5 | 2.6 (2.2) | 2.6 (2.2) | 2.6 (2.2) |  |
| **31,52,53,62** | α9, α9, α6, α3 | 7 | 2.2 (2.1) | 2.2 (2.2) | 2.2 (2.2) |  |
| **39,51,66,89** | α7, α5, α6, α3 | 6 | 2.2 (2.0) | 2.3 (2.1) | 2.3 (2.2) |  |
| **16,54,59,89** | α9, α13, α7, α3 | 6 |  |  | 2.4 (2.2) |  |
| **16,59,61,89** | α9, α7, α3, α3 | 6 |  |  | 2.3 (2.2) |  |
| **16,52,61,62** | α9, α9, α3, α3 | 8 |  |  | 2.1 (2.2) |  |
| **16,18,73,83** | α9, α7, α11, α3 | 5 | 3.8 (3.2) | 4.2 (3.5) | 2.5 (2.1) |  |
| **42,52,53,83** | α1, α9, α6, α3 | 6 | 3.1 (2.9) | 3.2 (3.1) | 2.3 (2.1) |  |
| **42,53,62,66** | α1, α6, α3, α6 | 7 | 2.9 (3.0) | 2.8 (2.8) | 2.2 (2.1) |  |
| **52,53,62,83** | α9, α6, α3, α3 | 7 | 2.7 (2.7) | 2.6 (2.7) | 2.2 (2.1) |  |
| **16,18,62,73** | α9, α7, α3, α11 | 5 | 2.6 (2.2) | 3.1 (2.7) | 2.5 (2.1) |  |
| **18,52,54,61** | α7, α9, α13, α3 | 5 | 2.7 (2.3) | 3.0 (2.6) | 2.5 (2.1) |  |
| **18,66,84,89** | α7, α6, α3, α3 | 5 | 2.6 (2.3) | 2.7 (2.3) | 2.5 (2.1) |  |
| **16,52,61,89** | α9, α9, α3, α3 | 7 |  |  | 2.2 (2.1) |  |
| **16,45,52,56** | α9, α7, α9, α6 | 5 |  |  | 2.5 (2.1) |  |
| **6,53,66,84** | α10, α6, α6, α3 | 5 |  |  | 2.5 (2.1) |  |
| **31,51,54,84** | α9, α5, α13, α3 | 5 |  |  | 2.5 (2.1) |  |
| **16,35,39,52** | α9, α9, α7, α9 | 7 |  |  | 2.2 (2.1) |  |
| **16,51,54,59** | α9, α5, α13, α7 | 7 |  |  | 2.1 (2.1) |  |
| **16,18,31,51** | α9, α7, α9, α5 | 6 |  |  | 2.3 (2.1) |  |
| **16,39,42,53** | α9, α7, α1, α6 | 7 |  |  | 2.2 (2.1) |  |
| **31,52,53,59** | α9, α9, α6, α7 | 6 |  |  | 2.3 (2.1) |  |
| **16,31,52,54** | α9, α9, α9, α13 | 7 |  |  | 2.2 (2.1) |  |
| **51,52,73,89** | α5, α9, α11, α3 | 5 | 3.2 (2.7) | 3.5 (3.0) |  |  |
| **42,51,56,89** | α1, α5, α6, α3 | 5 | 3.5 (3.0) | 3.4 (2.9) |  |  |
| **42,52,53,56** | α1, α9, α6, α6 | 6 | 3.0 (2.9) | 3.1 (2.9) |  |  |
| **6,53,56,89** | α10, α6, α6, α3 | 5 | 3.3 (2.8) | 3.4 (2.9) |  |  |
| **16,54,68,83** | α9, α13, α7, α3 | 5 | 3.3 (2.8) | 3.4 (2.9) |  |  |
| **52,53,54,73** | α9, α6, α13, α11 | 5 | 3.3 (2.9) | 3.3 (2.8) |  |  |
| **35,42,53,62** | α9, α1, α6, α3 | 5 | 3.2 (2.7) | 3.3 (2.8) |  |  |
| **56,59,66,89** | α6, α7, α6, α3 | 5 | 3.0 (2.6) | 3.3 (2.8) |  |  |
| **42,52,62,83** | α1, α9, α3, α3 | 5 | 3.0 (2.6) | 3.2 (2.8) |  |  |
| **42,52,56,62** | α1, α9, α6, α3 | 5 | 3.0 (2.6) | 3.2 (2.7) |  |  |
| **42,52,56,66** | α1, α9, α6, α6 | 5 | 3.3 (2.9) | 3.0 (2.6) |  |  |
| **42,51,62,83** | α1, α5, α3, α3 | 5 | 3.2 (2.7) | 3.0 (2.6) |  |  |
| **39,52,54,83** | α7, α9, α13, α3 | 5 | 2.9 (2.5) | 3.0 (2.6) |  |  |
| **42,51,62,66** | α1, α5, α3, α6 | 6 | 2.7 (2.5) | 2.7 (2.5) |  |  |
| **51,53,54,59** | α5, α6, α13, α7 | 7 | 2.5 (2.5) | 2.5 (2.5) |  |  |
| **42,51,54,59** | α1, α5, α13, α7 | 5 | 2.6 (2.2) | 2.9 (2.5) |  |  |
| **6,42,53,62** | α10, α1, α6, α3 | 5 | 2.9 (2.5) | 2.8 (2.4) |  |  |
| **42,51,53,61** | α1, α5, α6, α3 | 6 | 2.5 (2.4) | 2.6 (2.4) |  |  |
| **52,53,73,84** | α9, α6, α11, α3 | 5 | 2.6 (2.2) | 2.8 (2.4) |  |  |
| **18,53,56,66** | α7, α6, α6, α6 | 5 | 3.0 (2.6) | 2.7 (2.3) |  |  |
| **56,62,66,89** | α6, α3, α6, α3 | 5 | 2.9 (2.5) | 2.7 (2.3) |  |  |
| **31,53,56,59** | α9, α6, α6, α7 | 5 | 2.7 (2.3) | 2.7 (2.3) |  |  |
| **42,51,59,89** | α1, α5, α7, α3 | 5 | 2.6 (2.2) | 2.7 (2.3) |  |  |
| **51,52,54,84** | α5, α9, α13, α3 | 7 | 2.3 (2.2) | 2.3 (2.3) |  |  |
| **39,51,54,84** | α7, α5, α13, α3 | 6 | 2.3 (2.1) | 2.5 (2.3) |  |  |
| **39,42,52,89** | α7, α1, α9, α3 | 5 |  | 2.7 (2.3) |  |  |
| **6,62,66,89** | α10, α3, α6, α3 | 5 | 2.8 (2.4) | 2.6 (2.2) |  |  |
| **51,52,56,66** | α5, α9, α6, α6 | 6 | 2.4 (2.2) | 2.4 (2.2) |  |  |
| **18,31,52,53** | α7, α9, α9, α6 | 6 | 2.4 (2.2) | 2.4 (2.2) |  |  |
| **31,42,52,62** | α9, α1, α9, α3 | 5 | 2.4 (2.0) | 2.6 (2.2) |  |  |
| **53,56,61,89** | α6, α6, α3, α3 | 5 | 2.8 (2.4) | 2.5 (2.1) |  |  |
| **51,52,53,55** | α5, α9, α6, α10 | 5 | 2.7 (2.3) | 2.5 (2.1) |  |  |
| **51,56,59,66** | α5, α6, α7, α6 | 5 | 2.6 (2.2) | 2.5 (2.1) |  |  |
| **52,53,54,84** | α9, α6, α13, α3 | 7 | 2.2 (2.2) | 2.2 (2.1) |  |  |
| **18,51,66,89** | α7, α5, α6, α3 | 5 | 2.4 (2.1) | 2.5 (2.1) |  |  |
| **51,53,59,66** | α5, α6, α7, α6 | 7 | 2.2 (2.1) | 2.1 (2.1) |  |  |
| **42,53,59,89** | α1, α6, α7, α3 | 5 | 2.4 (2.0) | 2.5 (2.1) |  |  |
| **52,59,66,84** | α9, α7, α6, α3 | 6 |  | 2.3 (2.1) |  |  |
| **52,53,59,62** | α9, α6, α7, α3 | 7 |  | 2.2 (2.1) |  |  |
| **52,53,58,89** | α9, α6, α9, α3 | 5 |  | 2.4 (2.1) |  |  |
| **51,56,84,89** | α5, α6, α3, α3 | 5 | 2.5 (2.1) | 2.4 (2.0) |  |  |
| **39,42,51,89** | α7, α1, α5, α3 | 5 | 2.4 (2.0) | 2.4 (2.0) |  |  |
| **51,52,54,56** | α5, α9, α13, α6 | 5 | 2.4 (2.0) | 2.4 (2.0) |  |  |
| **52,56,66,84** | α9, α6, α6, α3 | 5 |  | 2.4 (2.0) |  |  |
| **16,56,59,89** | α9, α6, α7, α3 | 6 |  | 2.3 (2.0) |  |  |
| **53,61,62,83** | α6, α3, α3, α3 | 5 | 2.6 (2.2) |  |  |  |
| **52,61,62,83** | α9, α3, α3, α3 | 5 | 2.5 (2.1) |  |  |  |
| **53,56,61,62** | α6, α6, α3, α3 | 5 | 2.5 (2.1) |  |  |  |
| **51,53,66,89** | α5, α6, α6, α3 | 7 | 2.2 (2.1) |  |  |  |
| **51,56,66,84** | α5, α6, α6, α3 | 5 | 2.4 (2.0) |  |  |  |
| **52,53,61,62** | α9, α6, α3, α3 | 7 | 2.1 (2) |  |  |  |
| **6,53,61,62** | α10, α6, α3, α3 | 5 | 2.4 (2) |  |  |  |
| **18,51,53,66** | α7, α5, α6, α6 | 6 | 2.2 (2.0) |  |  |  |
| **42,53,66,89** | α1, α6, α6, α3 | 5 | 2.4 (2.0) |  |  |  |

Results are listed for HPV combinations with fdr ≤ 0.05. All 4-way associations were observed more than expected; no under observed 4-way combinations were significant. *HR-HPV types are underlined.
