# Supplementary material for: Randomization Modeling to Ascertain Clustering Patterns of Human Papillomavirus Types Detected in Cervicovaginal Samples in the United States
Source: PLoS One. 2013 Dec 18;8(12):e82761. doi: 10.1371/journal.pone.0082761 (PMC3867389; doi:10.1371/journal.pone.0082761)
Supplement: Table S5 — Number of times HPV types appeared in significant combinations. (DOC) [file pone.0082761.s008.doc]

Table S5. Number of times HPV types appeared in significant combinations.

|  |  | | | | | | | | | | | |
| --- | --- | --- | --- | --- | --- | --- | --- | --- | --- | --- | --- | --- |
|  |  | | |  | | |  | | |  | | |
|  | **Non-Strata** | | | **Study Strata** | | | **k Strata** | | | **Study-k Strata** | | |
| **HPV Types in Combination** | **2** | **3** | **4** | **2** | **3** | **4** | **2** | **3** | **4** | **2** | **3** | **4** |
| **HPV** |  |  |  |  |  |  |  |  |  |  |  |  |
| **6** | 1 | 7 | 27 | 1 | 5 | 26 |  |  | 25 |  |  | 9 |
| **11** | 1 | 6 |  |  | 5 |  |  |  |  |  |  |  |
| **16** |  | 3 | 31 |  | 2 | 32 | 2 | 3 | 60 | 1 |  | 9 |
| **18** | 1 | 5 | 22 |  | 3 | 20 | 1 | 1 | 22 | 1 |  | 4 |
| **26** | 1 | 2 |  |  | 1 |  |  |  |  |  |  |  |
| **31** |  | 3 | 35 |  | 2 | 35 | 1 | 1 | 45 | 1 |  | 16 |
| **33** | 1 | 2 | 1 | 1 | 2 | 1 | 1 | 1 | 1 | 1 | 1 |  |
| **35** | 2 | 11 | 10 |  | 9 | 12 |  |  | 14 |  |  | 6 |
| **39** |  | 5 | 27 |  | 4 | 29 | 1 | 3 | 35 |  | 1 | 8 |
| **40** |  | 25 | 2 |  | 19 | 2 |  | 1 | 2 |  |  |  |
| **42** | 2 | 19 | 53 |  | 17 | 53 |  |  | 41 |  |  | 9 |
| **45** |  | 1 | 4 |  | 1 | 4 | 1 | 2 | 6 | 1 |  | 2 |
| **51** | 4 | 16 | 65 | 4 | 11 | 63 | 3 | 2 | 54 | 3 |  | 8 |
| **52** | 1 | 7 | 54 | 1 | 6 | 57 | 1 | 1 | 53 | 1 | 1 | 6 |
| **53** |  | 7 | 75 |  | 3 | 69 |  |  | 58 |  |  | 16 |
| **54** | 1 | 9 | 36 |  | 6 | 36 |  | 3 | 32 |  |  | 8 |
| **55** | 1 | 12 | 9 | 1 | 5 | 9 | 1 | 1 | 8 | 1 |  | 6 |
| **56** | 2 | 22 | 66 | 1 | 17 | 64 | 1 | 5 | 51 | 1 | 3 | 23 |
| **58** | 2 | 4 | 17 | 2 | 2 | 17 | 2 | 1 | 18 | 2 | 1 | 4 |
| **59** |  | 3 | 39 |  | 3 | 45 |  | 1 | 43 |  |  | 10 |
| **61** | 3 | 16 | 27 |  | 3 | 23 | 4 | 5 | 27 |  |  | 10 |
| **62** | 4 | 18 | 37 | 2 | 7 | 35 | 6 | 7 | 28 | 2 | 1 | 12 |
| **64** |  |  |  |  |  |  |  |  |  |  |  |  |
| **66** | 3 | 24 | 75 | 3 | 18 | 71 | 1 | 6 | 64 | 1 | 3 | 28 |
| **67** |  | 6 | 1 |  | 4 | 1 | 1 | 1 | 1 |  |  | 1 |
| **68** |  | 8 | 6 |  | 7 | 6 |  | 2 | 4 |  |  | 3 |
| **69** | 1 |  |  | 1 |  |  | 1 |  |  | 1 |  |  |
| **70** |  | 5 | 2 |  | 1 | 2 | 3 | 5 | 2 |  | 1 | 1 |
| **71** | 2 | 6 |  |  | 3 |  | 3 |  |  |  |  |  |
| **72** | 2 | 1 |  |  |  |  | 4 |  |  |  |  |  |
| **73** | 1 | 13 | 25 |  | 10 | 25 |  |  | 23 |  |  | 3 |
| **81** | 2 | 9 | 3 |  | 1 | 3 | 2 | 2 | 3 | 1 |  | 2 |
| **82** | 1 | 8 | 1 | 1 | 5 | 1 | 1 | 3 | 1 | 1 |  | 1 |
| **83** | 6 | 19 | 29 | 1 | 8 | 26 | 5 | 5 | 22 | 2 | 1 | 6 |
| **84** |  | 12 | 30 |  | 8 | 30 | 1 | 3 | 28 |  | 1 | 12 |
| **89** | 2 | 13 | 67 | 2 | 9 | 67 |  | 4 | 61 |  | 1 | 21 |
| **IS39** | 1 |  |  | 1 |  |  | 1 |  |  | 1 |  |  |

The table summarizes how many times each HPV type appeared in significant (fdr ≤ 0.05) 2, 3 and 4 type combinations.
