# Supplementary material for: Randomization Modeling to Ascertain Clustering Patterns of Human Papillomavirus Types Detected in Cervicovaginal Samples in the United States
Source: PLoS One. 2013 Dec 18;8(12):e82761. doi: 10.1371/journal.pone.0082761 (PMC3867389; doi:10.1371/journal.pone.0082761)
Supplement: Methods S1 — Supplementary methods for statistical calculations. (DOC) [file pone.0082761.s009.doc]

**Methods S1.**  **Supplementary methods for statistical calculations.**

**Statistical Analysis**

*Expected Models*

To determine the expected number of concurrent infections, the matrix of observed results was randomized in five different ways, depending on the characteristics of the observed data being controlled, using R (Figure 1 and Table S2) [1]. Observed data had rows by subject and columns by 37 HPV types with zeros (0s) indicating negative HPV results and ones (1s) indicating positive (Figure 1A). For the null model of complete random association of HPV types in Fig. 1B, the 0s and 1s were shuffled within each column using the ‘sample’ function of the R *base* package version 2.14.1, preserving the count of each HPV type (fixed column sums) while allowing the number of types per subject to vary (variable row sums).

For the subsequent matrix randomizations, the ‘permatswap’ function created a series of naïve models, each with increasing fidelity to the real data, where some of the higher order data structure is preserved during the randomization [2]. The trial-swap method overcomes the deficiencies of other fixed column/row sum algorithms in avoiding biased randomized matrices [3]. The number of randomization steps was set to 5x10^7^ for the ‘burnin’ parameter as this value maximized Bray-Curtis dissimilarity values, indicating effective matrix randomization [3]. In the non-strata naïve model, the matrix was randomized while preserving the marginal HPV type counts and types per person (fixed row and column sums; Fig. 1C). For the next permutation models, observations were stratified based on the 6 studies (study strata), 1 through 14 concurrent infections (k strata), or a combination of both (study-k strata) using the ‘strata’ parameter of ‘permatswap’. Randomization within each strata had fixed column and row sums (Fig. 1D). To assess general trends of concurrent infections, 10,000 randomized matrices were generated from all the subjects (HPV positive and negative) and compared to the expected results. To assess the significance of specific type combinations, 1,000 randomized matrices were generated from the HPV positive subjects and analyzed as discussed below.

*Perl scripts for counting type combinations*

Counting occurrences of type combinations, whether in the observed data or in the randomized matrices for a given model, was done in Perl (Active Perl 5.8; ActiveState, Vancouver, BC). First each type combination was counted. Counts were stored in a “hash” data structure (associative array) with a 37 digit binary key (one digit for each HPV type). Then each of these keys were themselves parsed into type combinations for a separate hashed data structure – e.g. two type combinations, three type combinations, etc. – using a modification of Knuth’s “n choose k” algorithm [4]. This was done so that specific combinations of HPV types could be counted irrespective of whether other types were present or not. Because the counting algorithm is machine resource intensive, and a typical high-end workstation (12 CPU cores and 12 Gb of RAM) often could not process all 1,000 matrices in a single run, the results of several smaller runs were combined using a different Perl script. The combined results for the randomized matrices were then matched to type combinations observed in the real data.

*Mathematica scripts for computing p-values and Z-scores*

Results for the Perl scripts above were then read into a Mathematica program to assess statistical significance (Wolfram Research, Champaign IL). First, the expected counts of any given type combination in the naïve models were fit to a Poisson probability density function (pdf; Figure S1A). Fits always conformed to a Poisson pdf with a model p-value ≥ 0.99 (done with the JMP 9.0 software, SAS Institute, Cary NC). Because a Poisson distribution has only one parameter, assessing the statistical significance of any given type combination in the real data depended only on knowledge of the mean value observed in the randomized matrices of the various naïve models. To graphically indicate significant type combinations, the observed counts were compared to p-value boundaries created from the permutation models. These boundary counts were created using a polynomial fit to several solutions of the number of observations expected from Poisson distributions with different mean values (Figure S1B – all fits were as good as shown or better) so that boundary values could be computed for evenly spaced points. Type combination counts corresponding to p-value boundaries of 10^-4^, 10^-6^, 10^-8^, etc. were calculated for both the right (observed > expected) and left (observed < expected) tails of the Poisson distribution. Observed counts were then plotted against expected counts for a given naïve model so that type combinations falling outside the boundary value lines could be easily seen. Besides the hypothetical p-value boundaries, actual p-values and Z-scores were computed for each specific type combination using the Poisson fit mean values from the Monte Carlo runs. Because the type combination frequencies observed in the 1,000 randomized matrices precisely fit a known and well characterized distribution, p-values < 0.001 can be reliably estimated.

*Multiple Hypothesis Testing Correction*

Due to the number of HPV type combinations analyzed, the Benjamini and Hochberg false-discovery rate (fdr) was calculated using ‘p.adjust’ in R to control for spurious results [5]. Rare observed type combinations with very low expected values were excluded from consideration by eliminating type combinations when both the observed and naïve model data had counts ≤ 4, e.g. observed = 4, expected = 0.1. With p-values skewed towards observations occurring more than expected, the fdr was calculated separately for the more than expected and the less than expected results in each null/naïve model.

All code (R, Perl, or Mathematica) is available upon request.

1. R Core Team (2011). R: A Language and Environment for Statistical Computing. R Foundation for Statisctical Computing, Vienna, Austria. ISBN 3-900051-07-0, URL http://www.r-project.org/.

2. Oksanen J, Blanchet FG, Kindt R, Legendre P, Minchin PR, et al. (2011) Vegan: Community Ecology Package. Version 2.0-1.

3. Miklós I, Podani J (2004) Randomization of Presence-Absence Matrices: Comments and New Algorithms. Ecology 85: 86-92.

4. Knuth DE (2005) Generating all combinations and partitions. In: The art of computer programming. Upper Saddle River (N.J.): Addison-Wesley.

5. Benjamini Y, Hochberg Y (1995) Controlling the false discovery rate: a practical and powerful approach to multiple testing. Journal of the Royal Statistical Society Series B 57: 289-300.
